# Friction Stability and Wear Behavior of Te NW–TiB_2_/Epoxy Composites

**DOI:** 10.3390/polym18192380

**Published:** 2026-09-29

**Authors:** Ahmet Sarucan, Hayrettin Düzcükoğlu, Bayram Ünal, Mehmet Emin Baysal, Mürsel Ekrem

**Affiliations:** 1 Department of Industrial Engineering, Konya Technical University, 42250 Konya, Türkiye; asarucan@ktun.edu.tr (A.S.); mebaysal@ktun.edu.tr (M.E.B.); 2 Department of Mechanical Engineering, Selçuk University, 42075 Konya, Türkiye; 3 Department of Metallurgical and Materials Engineering, Selçuk University, 42075 Konya, Türkiye; 4 Department of Mechanical Engineering, Necmettin Erbakan University, 42075 Konya, Türkiye

**Keywords:** CRITIC weighting, dry sliding friction and wear, epoxy hybrid composite, tellurium nanowires, Te NW, TiB_2_, multi-criteria decision-making (MCDM)

## Abstract

Epoxy thermosets offer excellent dimensional stability and mechanical rigidity, but their inherent brittleness limits their durability in dynamic sliding applications. To overcome this limitation, we synthesized a multi-dimensional hybrid filler composed of one-dimensional Tellurium Nanowires (Te NWs) and Titanium Diboride (TiB_2_) particles via an in situ hydrothermal method to improve the dry-sliding friction and wear resistance of the epoxy matrix. Neat epoxy and nanocomposites containing 0.25–1.00 wt.% hybrid filler were tested against hardened AISI 8620 steel over a sliding distance of 2000 m at normal loads of 5–10 N and sliding velocities of 1.00–1.75 m/s. Tribological stability and wear behavior were systematically assessed using the mean coefficient of friction (COF), dispersion metrics (standard deviation, coefficient of variation, and interquartile range), percentage mass loss, infrared thermography, and field-emission scanning electron microscopy (FESEM) of the worn surfaces. The hybrid composites showed a nonlinear tribological response to filler loading. The 0.50 wt.% Te NW–TiB_2_/epoxy composite delivered the best overall performance, particularly under the most demanding conditions (10 N and 1.75 m/s). It achieved the lowest mean COF (0.181 ± 0.027), minimal percentage mass loss (0.12%), a narrow friction distribution (coefficient of variation ≤ 13.61%; interquartile range ≤ 0.027), and a lower maximum surface temperature measured by infrared thermography (54.1 °C, compared with 68.6 °C for neat epoxy). Criterion weights were calculated using the Criteria Importance Through Intercriteria Correlation (CRITIC) method and validated using the entropy method. Across all four multi-criteria decision-making methods, the 0.50 wt.% formulation consistently ranked highest, identifying it as the best-performing hybrid composition within the tested range, with 0.75 wt.% as a close alternative.

## 1. Introduction

Friction and wear are major sources of energy loss and material consumption in engineering systems. Recent advances in science and engineering have made it increasingly important to optimize the friction and wear characteristics of dynamic components [1]. Epoxy resin is widely used in industries such as automotive manufacturing, aerospace structures, and packaging because of its high mechanical integrity, minimal volume shrinkage, and strong chemical resistance [2]. However, its dense, cross-linked network structure makes epoxy brittle, with low toughness and limited wear resistance. These drawbacks restrict its use in dynamic machine components such as bearings, bushings, gears, cams, and slides, where friction and wear are critical concerns [3]. Filler materials used in tribological epoxy composites have diverse properties. Hard ceramic particles, including silicon carbide (SiC), aluminum oxide (Al_2_O_3_), titanium dioxide (TiO_2_), and TiB_2_, help support contact loads, reduce matrix deformation, and improve hardness and wear resistance. These composites also incorporate low-shear-resistance solid lubricants, such as graphite, molybdenum disulfide (MoS_2_), and hexagonal boron nitride (h-BN). These lubricants promote the formation of shear layers and transfer films, significantly reducing friction [4,5,6]. Hybrid filler systems that combine high-hardness reinforcement phases with low-shear-resistance phases have attracted considerable interest for their synergistic properties, enabling both high load-carrying capacity and reduced friction [7].

The geometric dimensionality and spatial architecture of functional fillers are key design parameters for optimizing the tribological performance of advanced polymer composites. Integrating multi-dimensional filler systems, including zero-dimensional (0D) nanoparticles, one-dimensional (1D) extended structures, and two-dimensional (2D) planar nanosheets, has been reported to provide complementary reinforcement effects that are difficult to obtain with a single filler type [8]. Under dry sliding conditions, 1D elements with high aspect ratios, such as nanotubes and nanowires, bridge propagating microcracks, stabilize subsurface slip zones, and distribute local contact stresses along their longitudinal axes. Simultaneously, dispersed 0D and 2D particles act as effective load-bearing asperities and solid lubricants, reducing direct matrix–counter surface interactions and limiting severe micro-ploughing [9]. In addition, local shear and frictional flash heating have been reported to promote the compaction and tribochemical evolution of multi-scale wear debris and the in situ formation of a tribofilm on the counterface. In such systems, the multi-dimensional filler network has been proposed to anchor the tribofilm, delay its removal, and assist heat dissipation from the real contact area, thereby stabilizing friction and reducing wear [10].

Adding small amounts of graphene oxide (GO; 0.3–0.5 wt.%) to epoxy (EP) composites consistently enhances their tribological performance by reducing localized heat buildup, increasing matrix stiffness, and promoting the formation of a uniform transfer layer. These effects reduce wear rates by 15–35% [11]. Yan et al. demonstrated that incorporating a hybrid graphene/Ti_3_C_2_T_x_ additive into an EP matrix reduced the wear rate by 81–88%. This reduction was attributed to the synergistic lubrication and load-bearing effects of the two-dimensional phases. These findings highlight the potential of two-dimensional, lamellar graphene/Ti_3_C_2_T_x_ MXene heterostructures as dual-functional additives that enhance both the mechanical stiffness and self-lubricating properties of epoxy systems. However, achieving robust interfacial bonding and homogeneous dispersion of these inorganic two-dimensional nanolayers within the organic epoxy network remains a significant challenge, limiting the full utilization of their intrinsic properties [12]. To address friction and wear limitations, a silane-bridged h-BN/MoS_2_ heterostructure was incorporated into EP. Surface-grafted h-BN nanolayers prevented oxidative degradation of the underlying MoS_2_ spheres, enabling continuous self-lubrication at the sliding interface. At an optimal loading of 5 wt.%, the h-BN/MoS_2_/EP composite exhibited superior tribological properties compared with EP, with an 88% reduction in the coefficient of friction (to 0.026) and a 99% reduction in the specific wear rate (to 1.5 × 10^−6^ mm^3^/Nm) [13].

Tellurium (Te) and its low-dimensional nanostructures have attracted considerable interest for functional applications, including thermoelectric conversion systems [14], optoelectronics [15], energy storage, and electrochemical biosensors [16]. Among one-dimensional nanomaterials, Te NWs are particularly promising owing to their trigonal, helical-chain crystal structure, high aspect ratio, pronounced structural anisotropy, and favorable axial transport properties [17]. Despite these notable physicochemical and electronic properties, the incorporation of elemental tellurium powder into polymer matrices for structural or surface-engineering applications remains largely unexplored. In particular, the friction and wear behavior, subsurface deformation response, and potential tribofilm-formation mechanisms of Te-reinforced polymer composites have not yet been investigated in the literature, which motivates the present study.

Previous studies have reported mechanical and thermal enhancements in Te NW-reinforced epoxies. Balguri et al. found that adding just 0.05 wt.% Te NWs (approximately 21 nm in diameter and 697 nm in length) increased flexural strength by approximately 65%, attributing the improvement to strong interfacial bonding and suitable linear alignment [18]. Yan et al. demonstrated that three-dimensional interconnected Te NW networks can enhance the thermal conductivity of epoxy [19].

TiB_2_ is a widely used ceramic reinforcement owing to its high hardness (approximately 25–30 GPa), high melting point, chemical inertness, and resistance to mechanical wear [20]. In engineering matrices, these properties enable TiB_2_ particles to act as hard, load-bearing surface features that resist micro-cutting, ploughing, and shear-induced degradation [21]. Chenrayan et al. demonstrated that an aluminum–matrix composite reinforced with a hybrid TiB_2_/graphene structure achieved an optimized wear coefficient under challenging conditions [22]. In polymeric systems, adding a hybrid TiC–TiB_2_ filler (20 wt.%) to epoxy/glass-fiber composites significantly improved wear and erosion resistance, reduced the coefficient of friction, and increased wear resistance by 8% [23]. Although incorporating ceramic phases such as TiC and TiB_2_ can reduce the matrix’s tensile and flexural strength by creating stress concentrations, studies have shown that suitable hybridization can restore or even enhance these mechanical properties [21].

The design and tribological evaluation of polymer composites containing Ti-based ceramic phases involve complex trade-offs among operational variables and conflicting response criteria, such as wear resistance, friction stability, and frictional heating [24]. While various optimization strategies, including response surface methodology (RSM), grey relational analysis (GRA), and metaheuristic algorithms, have been used to address these multivariate domains [25,26], traditional regression-based models often struggle with complex nonlinear relationships in simultaneous multiple-response optimization [27]. To overcome these challenges, mathematically robust, objective weighting frameworks that derive criterion significance directly from the structure of the experimental data are necessary. Objective multi-criteria decision-making (MCDM) models using CRITIC and Shannon entropy weighting have been applied to resolve conflicting tribological responses. The CRITIC method incorporates both standard deviation and correlation to address conflicts between features. In contrast, the entropy method quantifies the data distribution and assigns greater analytical weights to criteria with strong discriminative power. Integrating these objective models with experimental evaluations enables an unbiased assessment of the performance of multi-filled composites [28].

This study investigated the fabrication of epoxy composites reinforced with TiB_2_ and one-dimensional Te nanowires, as well as their tribological behavior under dry sliding conditions. The study compared the effects of filler ratio, normal load, and sliding speed on the mean COF, COF distribution, mass loss, and maximum surface temperature, as measured by infrared thermography. The morphological properties of worn composite surfaces were evaluated using FESEM images and correlated with tribological results. Possible wear and heat-transfer mechanisms related to the findings were discussed, considering the limitations of the available experimental evidence; worn-surface images were not used as evidence of volumetric nanowire percolation. Furthermore, the CRITIC and Shannon entropy weighting methods, along with TOPSIS, VIKOR, WASPAS, and GRA, were applied to rank the formulations and identify the best-performing filler ratio among those tested.

## 2. Materials and Methods

### 2.1. Materials

K_2_TeO_3_, D-glucose, and TiB_2_ were procured from Merck, NJ, USA. The epoxy resin (MGS-L285) and hardener (MGS-H285) were obtained from Momentive, Niskayuna, NY, USA. At the recommended weight ratio of 100:40, the material’s gelation time was estimated to be 2–3 h at room temperature.

### 2.2. Tellurium Nanowire and In Situ Te NW–TiB_2_ Synthesis

**Figure 1:**
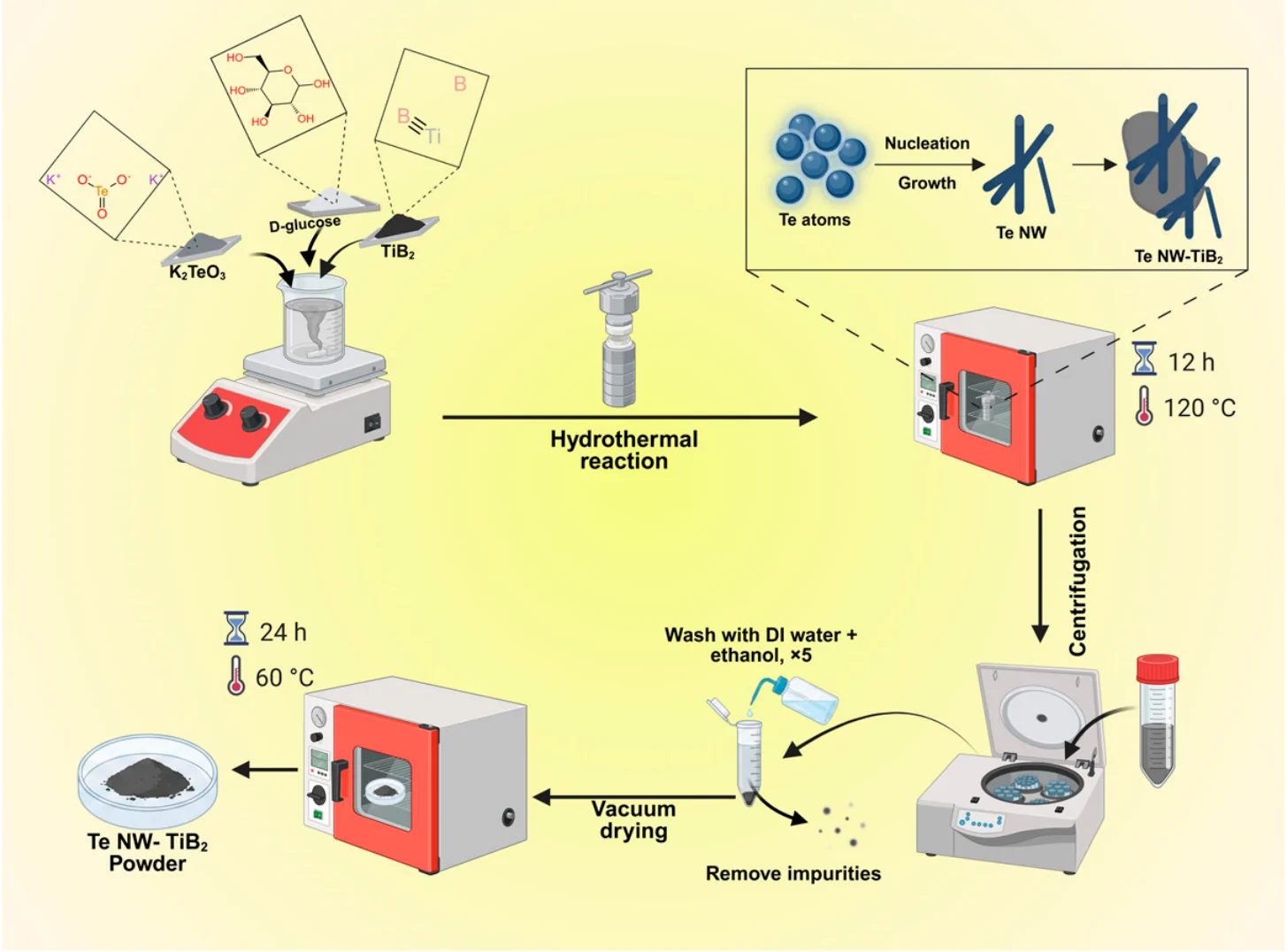
Te NW–TiB_2_ Synthesis Procedure.

One-dimensional tellurium nanowires (Te NWs) were synthesized using a green hydrothermal method adapted from Xu et al. [29]. First, 0.1203 g of K_2_TeO_3_ and 2.9904 g of D-glucose were dissolved in 60 mL of deionized water, and 0.200 g of TiB_2_ powder was added. The mixture was reacted in a 100 mL Teflon-coated autoclave at 120 °C for 12 h. After cooling to room temperature, the product was centrifuged at 5000 rpm, washed five times with water and ethanol, and dried under vacuum at 60 °C for 24 h (Figure 1). Complete reduction of the 0.1203 g K_2_TeO_3_ precursor would theoretically yield a maximum of 0.0605 g of elemental Te. Assuming complete conversion and the addition of 0.200 g of TiB_2_, the total theoretical product mass is 0.2605 g (approximately 23% Te and 77% TiB_2_ by mass). It should be noted that the final hybrid filler composition and overall synthesis yield were not quantitatively assessed, and actual post-synthesis ratios may vary due to washing and centrifugation steps. Allowing for typical losses during hydrothermal synthesis and centrifugation, the practical powder yield is estimated at 0.230–0.255 g; this estimate was not verified experimentally.

### 2.3. Fabrication of Te NW–TiB_2_-Reinforced Epoxy Nanocomposites

**Figure 2:**
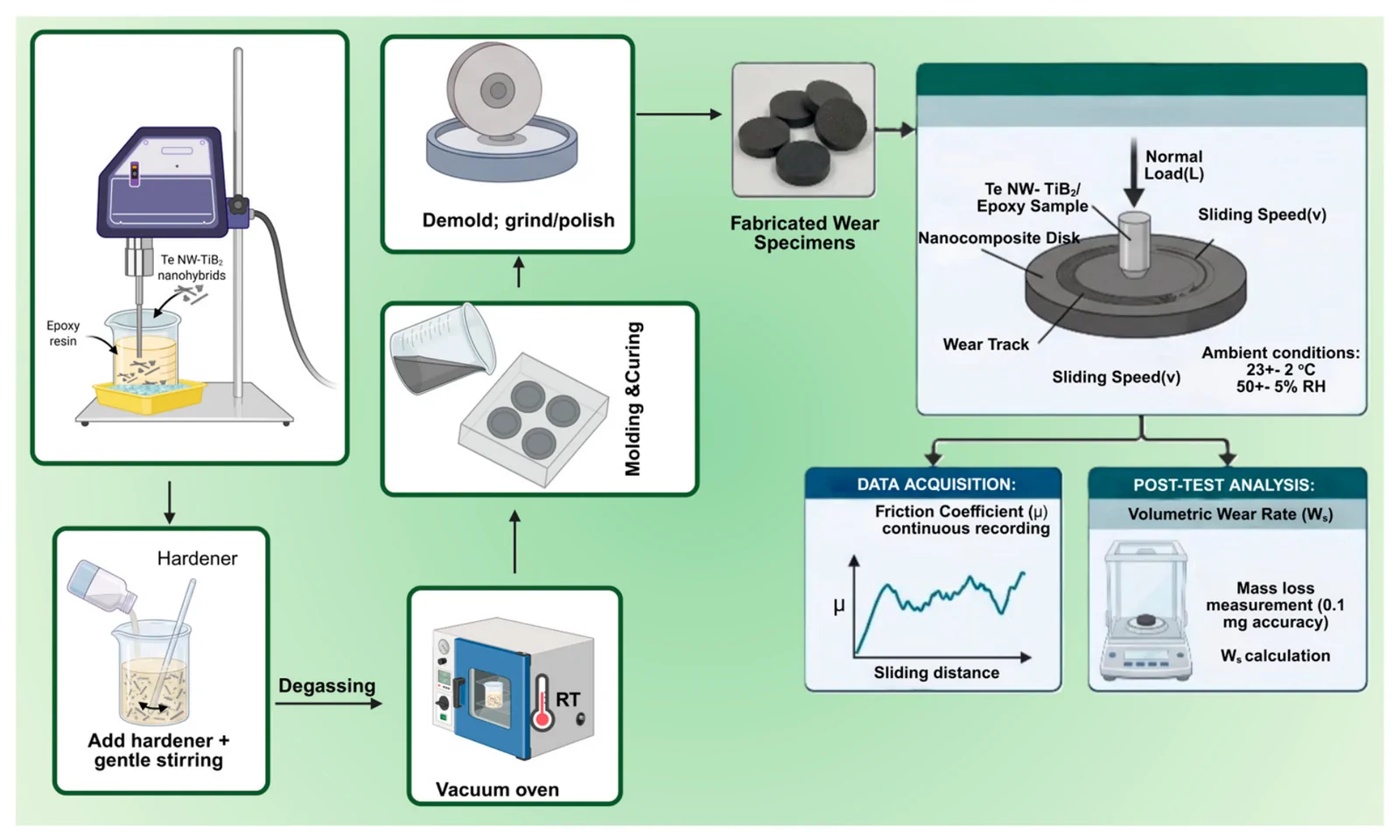
Workflow for specimen fabrication and tribological characterization of Te NW–TiB_2_/Epoxy Composites.

To ensure uniform filler distribution and prevent localized agglomeration, Te NW–TiB_2_ nanostructures were incorporated into the epoxy resin by probe sonication. Nanohybrids were added at concentrations of 0.25, 0.50, 0.75, and 1.0 wt.%. The mixtures were processed using a probe-type ultrasonic homogenizer (Bandelin HD 2070, Germany, 20 kHz, KE 76 probe tip) at 35% amplitude (~70 W). Sonication was carried out for 20 min in 5 min pulsed cycles [30]. The beaker was kept in an ice-water bath during sonication to prevent excessive heat buildup and localized pre-curing. All mixing and sonication parameters were kept constant across batches, and the equipment was calibrated before each run. A stoichiometric amount of hardener was then incorporated into the suspension by gentle mechanical mixing to further homogenize the mixture. The mixture was subsequently degassed in a vacuum oven at room temperature to remove trapped air bubbles, then poured into silicone molds designed specifically for wear-test specimens. In tribological experiments, cylindrical composite pins with a diameter of 10 mm and a length of 15 mm were used; the 10 mm-diameter flat contact surface provided a nominal contact area of approximately 78.5 mm^2^. Following the proposed curing and post-curing protocols, the demolded specimens were sanded and polished using SiC abrasive papers (up to 2000 grit) to remove surface defects and achieve a consistent initial surface roughness (Ra). All specimens of a given filler loading were prepared using the same processing parameters. The preparation procedures for the epoxy nanocomposites are shown in Figure 2.

### 2.4. Characterization of Materials

The morphological, surface topographic, and fractographic properties of the Te NW–TiB_2_ hybrid composite and epoxy surface were examined using field-emission scanning electron microscopy (FESEM; ZEISS EVO LS10, Jena, Germany) at an accelerating voltage of 2 kV. Before analysis, the samples were coated with a 4.73 nm iridium layer for 20 min under high-vacuum conditions to reduce surface charging and enhance secondary-electron emission. The crystal structure, phase purity, and structural transitions of the synthesized Te NW–TiB_2_ nanocomposites were characterized by X-ray diffraction (XRD; Panalytical Empyrean, Almelo, Netherlands) over a 2θ range of 10–80° using Cu Kα radiation (λ = 0.154 nm).

### 2.5. Wear Tests

Dry sliding wear tests were conducted in accordance with ASTM G99-23 [31] at an ambient temperature of 23 ± 2 °C and relative humidity of 35 ± 5% using a standard pin-on-disc tribometer. AISI 8620 steel discs, 15 mm thick and with a hardness of 56 HRC, served as the counter surface. A consistent wear track with a diameter of 52 mm (26 mm radius) was used in all experiments. A new steel disc was used for each experimental condition to prevent surface changes and material transfer from previous tests. Before testing, the composite pins were polished with SiC abrasive paper up to 2000 grit, and the AISI 8620 steel discs were ground. The surface roughness of both contact partners was measured using a Mitutoyo Surftest SJ-210 stylus surface roughness tester (model 178-560-01D, Kawasaki, Japan) with a cut-off length (λc) of 0.8 mm, an evaluation length of 4 mm, and a measuring speed of 0.5 mm/s. Measurements were taken at three different locations on each surface perpendicular to the polishing/grinding marks. The average surface roughness (mean ± standard deviation) was Ra = 0.40 ± 0.05 µm for the composite pins and Ra = 0.15 ± 0.03 µm for the steel discs. Before each test, the disc surfaces were cleaned with methanol and left in the laboratory for approximately 1 min to allow the solvent to evaporate completely. The test system was powered by a 2.2 kW motor with a nominal speed of 1400 rpm and an operating frequency of 50 Hz. At sliding speeds of 1.00, 1.50, and 1.75 m/s, the disc rotation speeds were approximately 367, 551, and 643 rpm, respectively, with drive frequencies set to 13.12, 19.68, and 22.96 Hz. Actual rotation speeds were verified using a PCE-DT 65 digital laser tachometer (Meschede, Germany). Friction force was measured using a bellows-type load cell (SBK) with a capacity of 10 kgf, and signals were recorded at a sampling rate of 50 Hz using a Squirrel SQ2020-2F8 data logger (Grant Instruments, Shepreth, UK). Each experimental condition was tested three times (R1–R3). The replicate tests were run consecutively and, in most cases, on the same pin specimen, so that the final mass of one replicate is the initial mass of the next (Table S1); a new steel disc was used for each experimental condition. The replicates therefore describe the repeatability of consecutive tests rather than fully independent specimen-to-specimen variability, and R2 and R3 started from a surface already worn in the preceding test. A fixed running-in distance was not defined because running-in behavior varied among experimental conditions. All COF data collected over sliding distances of 0–2000 m were evaluated using the SD, CV, median, and IQR. Specimens were weighed before and after each sliding test using an AS 220.R2 PLUS analytical balance (Radom, Poland) with a readability of 0.1 mg. The initial and final masses, absolute mass losses (Δm), and percentage mass losses of every replicate specimen are reported in Table S1 (Supplementary Materials); along with the means of the three replicates for each condition. No certified measurement-uncertainty analysis was performed for the balance; the reliability of the mass-loss data is therefore described by the balance readability (0.1 mg) and by the replicate-to-replicate scatter reported in Table S1. Absolute mass loss (Δm) and percentage mass loss were calculated using Equations (1) and (2), respectively [32]:

(1) $$\Delta m=m_{i}-m_{f}$$

(2) $$\mathrm{Percentage}\;\mathrm{mass}\;\mathrm{loss}\;=\frac{m_{i}-m_{f}}{m_{i}}\times 100\%$$

where $m_{i}$ and $m_{f}$ represent the specimen masses before and after the sliding test, respectively. Accordingly, the reported values represent percentage mass loss, not specific or volumetric wear rates. Percentage mass loss is the ratio of the material removed during a sliding test to the sample’s initial mass. This normalized measure enables comparisons between samples with slightly different initial masses; lower values indicate greater resistance to material loss under the applied test conditions.

The surface temperature of the pin–disc contact region was measured using a Raythink CX200 series handheld infrared thermal camera (Teknotherm, Turkey). The camera is equipped with a 256 × 192-pixel uncooled infrared detector with a 12 µm pixel pitch, a spectral range of 7.5–14 µm, a frame rate of 25 Hz, a 56° × 42° field of view, and an IFOV of 3.75 mrad. Its temperature measurement accuracy is ±2 °C. The camera was used within the validity of the manufacturer’s calibration certificate. For the relatively matte, non-metallic epoxy composite surface, emissivity (ε) was set to 0.95 and kept constant across all experiments. The camera was mounted on a tripod approximately 25 cm from the pin–disc contact region, at an angle of approximately 15° to the disc surface. Thermal images were recorded when the sliding distance reached approximately 1750 m; thus, temperature was not monitored continuously throughout the experiments. The region of interest (ROI) was defined as the area encompassing the visible contact perimeter of the pin and rotating disc. For each thermogram, the highest pixel temperature within the ROI was taken as the maximum surface temperature using the device’s highest-temperature-point determination function.

Each experimental condition was tested in three replicates (see above). For each replicate, the mean COF, SD, CV, and IQR were calculated separately from instantaneous COF values over the 0–2000 m range. Inter-experimental repeatability was assessed using the overall mean ± standard deviation of the mean COF values across the three replicates. Because an objective plateau could not be identified across all curves, no fixed running-in distance was assigned. Results are therefore reported for the full sliding interval (0–2000 m) rather than for a ‘steady state’.

## 3. Results

### 3.1. FESEM Analysis of Te NW–TiB_2_

**Figure 3:**
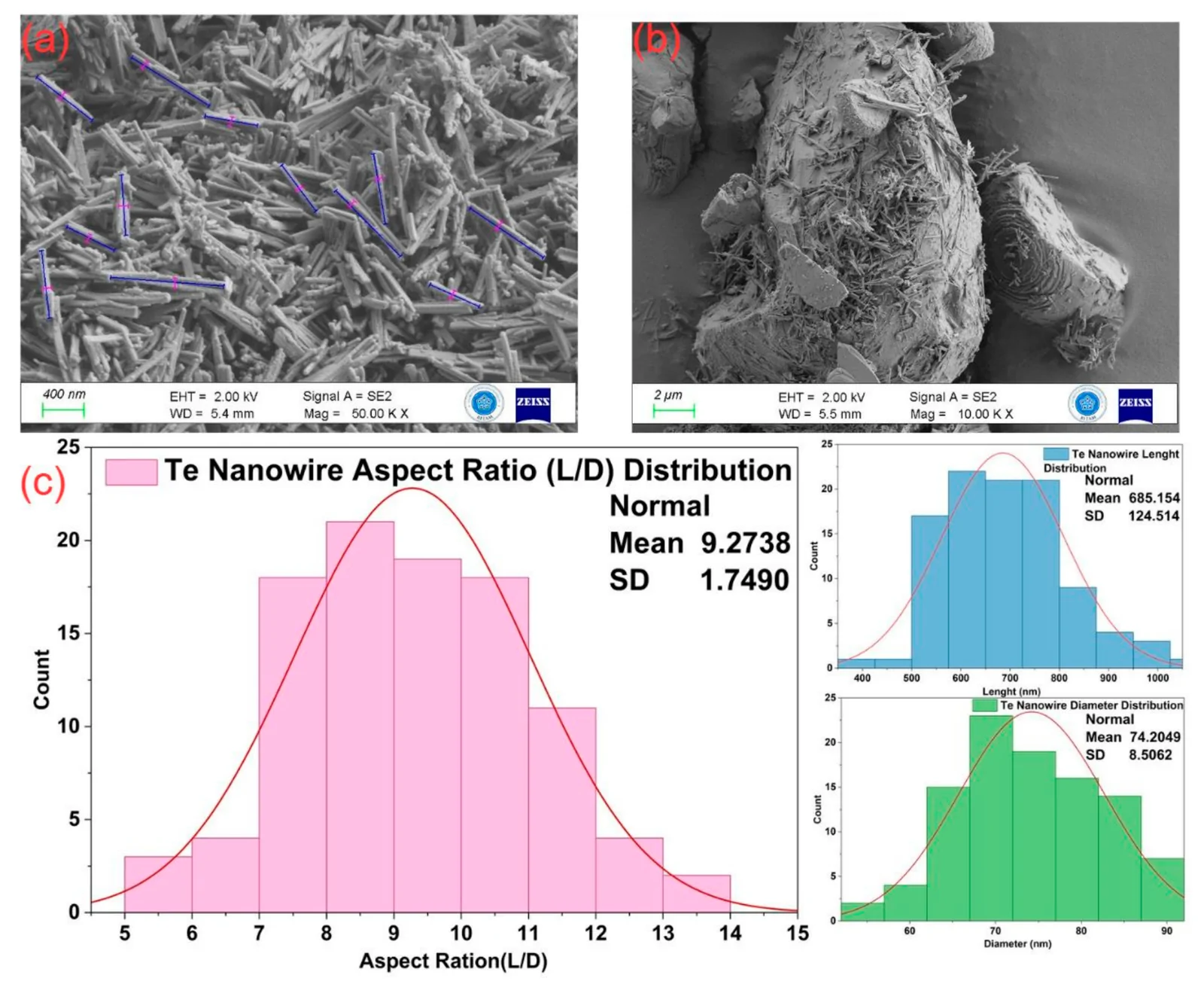
(**a**) Tellurium Nanowires; (**b**) in situ-synthesized Te NW–TiB_2;_ (**c**) Size distribution.

Figure 3a,b show FESEM micrographs of the synthesized Te NWs and the in situ-synthesized Te NW–TiB_2_ hybrid powders, respectively. As seen in Figure 3a, the nanowires extend randomly, displaying localized overlapping and bundling; the blue and pink lines mark representative length and diameter measurements, respectively. Figure 3b demonstrates the morphological integration of the Te NWs around the TiB_2_ particles, consistent with the formation of the hybrid architecture via the in situ hydrothermal route.

Figure 3c presents histograms of the Te nanowire length, diameter, and length-to-diameter ratio (L/D). The mean length is 685.15 ± 124.51 nm, the mean diameter is 74.20 ± 8.51 nm, and the mean L/D is 9.27 ± 1.75 (mean ± standard deviation). The horizontal axes show the respective dimension parameters, and the vertical axes show the number of measurements in each class interval. L/D is dimensionless and denotes the ratio of nanowire length to diameter. The relative spreads of the length and L/D distributions exceed that of the diameter distribution; thus, the diameters in the sample studied have a relatively narrower distribution.

### 3.2. XRD Analysis

**Figure 4:**
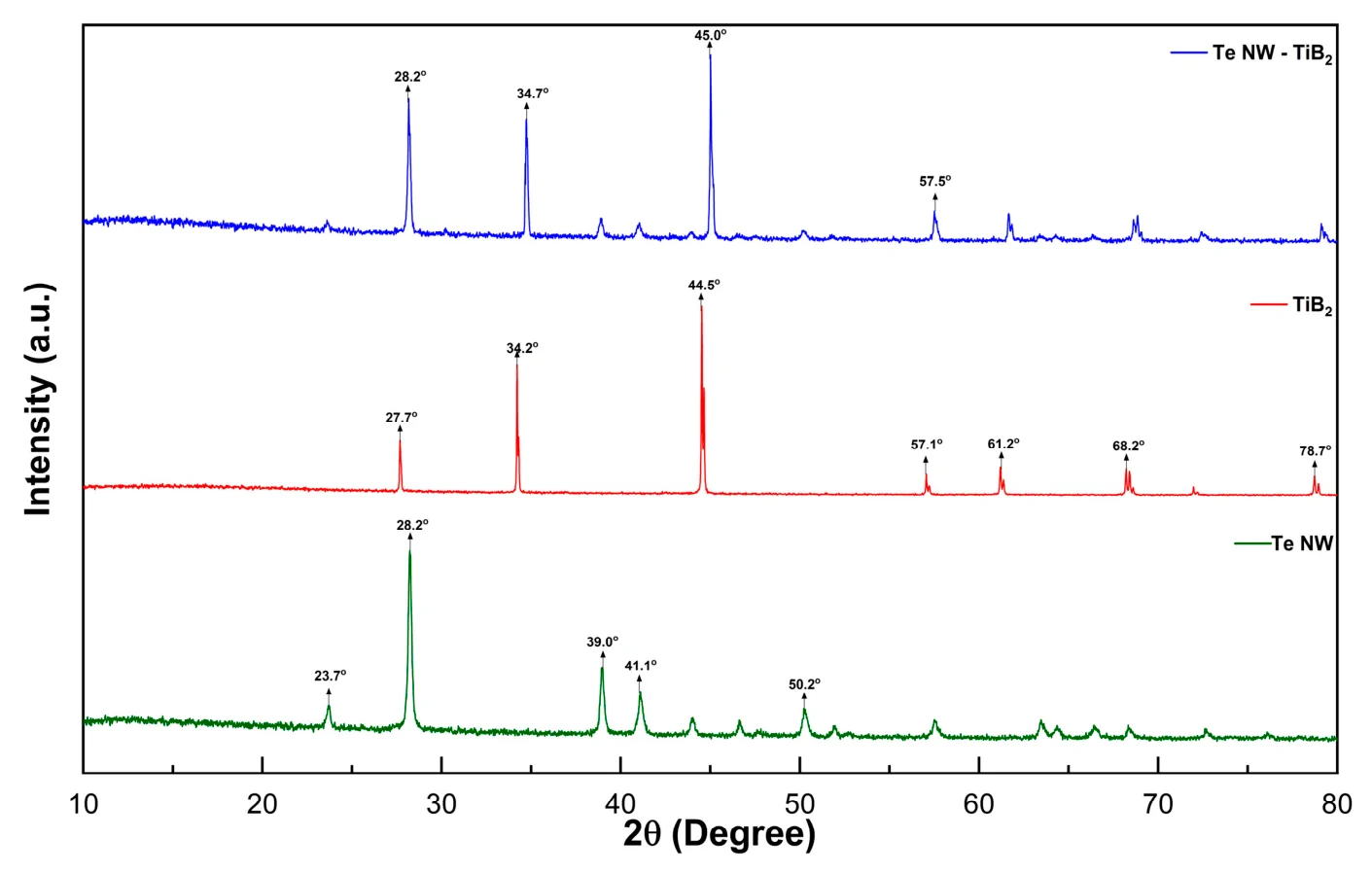
X-ray diffraction (XRD) patterns of Te NW, and Te NW-TiB_2_.

The crystalline phases of the synthesized Te NW, TiB_2_, and Te NW–TiB_2_ composite were examined using the X-ray diffraction (XRD) patterns shown in Figure 4. The TiB_2_ reflections are consistent with a hexagonal crystal structure (space group P6/mmm; JCPDS Card No. 35-0741). Peaks at approximately 27.7°, 34.2°, 44.5°, 57.1°, 61.2°, 68.2°, and 78.7° 2θ were assigned to the (001), (100), (101), (002), (110), (102), and (201) planes of TiB_2_, respectively. The prominent (101) reflection at 44.5° is consistent with the presence of crystalline TiB_2_. The Te NW diffraction pattern was associated with trigonal tellurium (t-Te; space group P3_1_21; JCPDS Card No. 36-1452). Characteristic peaks at approximately 23.7°, 28.2°, 39.0°, 41.1°, and 50.2° 2θ were assigned to the (100), (011), (102), (110), and (201) planes, respectively. The prominent reflection at approximately 28.2° is consistent with crystalline Te. The hybrid filler pattern contains reflections assigned to both Te and TiB_2_, supporting the coexistence of the two crystalline phases. Prominent peaks in the hybrid pattern occur around 28.2° for Te and 34.7°, 45.0°, and 57.5° for TiB_2_. The slight peak displacements (approximately 0.4–0.5° 2θ) are commonly associated with experimental sample surface height discrepancies and localized defect-induced lattice relaxation during synthesis. No significant additional diffraction peaks attributable to oxide phases, such as TiO_2_, B_2_O_3_, or TeO_2_, or to residual synthesis precursors were observed across the examined 10–80° 2θ range. However, the absence of such peaks does not exclude minor phases below the XRD detection limit or weakly crystalline or amorphous species.

### 3.3. Tribological Properties of Te NW–TiB_2_/Epoxy Composites

Evaluating polymer-composite tribological performance solely by the mean coefficient of friction (μ) is insufficient to fully characterize dynamic contact-interface regimes [33]. Although lower friction indicates reduced resistance to shear, it does not necessarily ensure low mass loss or the formation of a continuous transfer film, because friction and wear arise from different physical mechanisms [34]. Accordingly, contact stability was quantitatively assessed using the mean COF (μ), standard deviation (SD), coefficient of variation (CV), and interquartile range (IQR), calculated using Equations (3)–(6), respectively.

μ = (1/n) ∑ μ_i_ (3)

(4) $$SD=\sqrt{\frac{\Sigma_{i=1}^{n}{\left(xi-\bar{x}\right)}^{2}}{n-1}}$$

(5) $$CV(\%)=\frac{SD}{\bar{\mu }}\times 100$$

IQR = Q_3_ − Q_1_ (6)

μ represents the instantaneous coefficient of friction, and n denotes the total number of data points examined over the 0–2000 m range. Q_1_ and Q_3_ denote the dataset’s 25th and 75th percentiles, respectively. Low SD, CV, and IQR values indicate a narrower distribution of COF values within the selected analysis range. These statistics alone do not establish the formation or degradation of the transfer film, matrix plastic deformation, or other physical wear mechanisms; they quantify only the variability of the friction signal.

**Figure 5:**
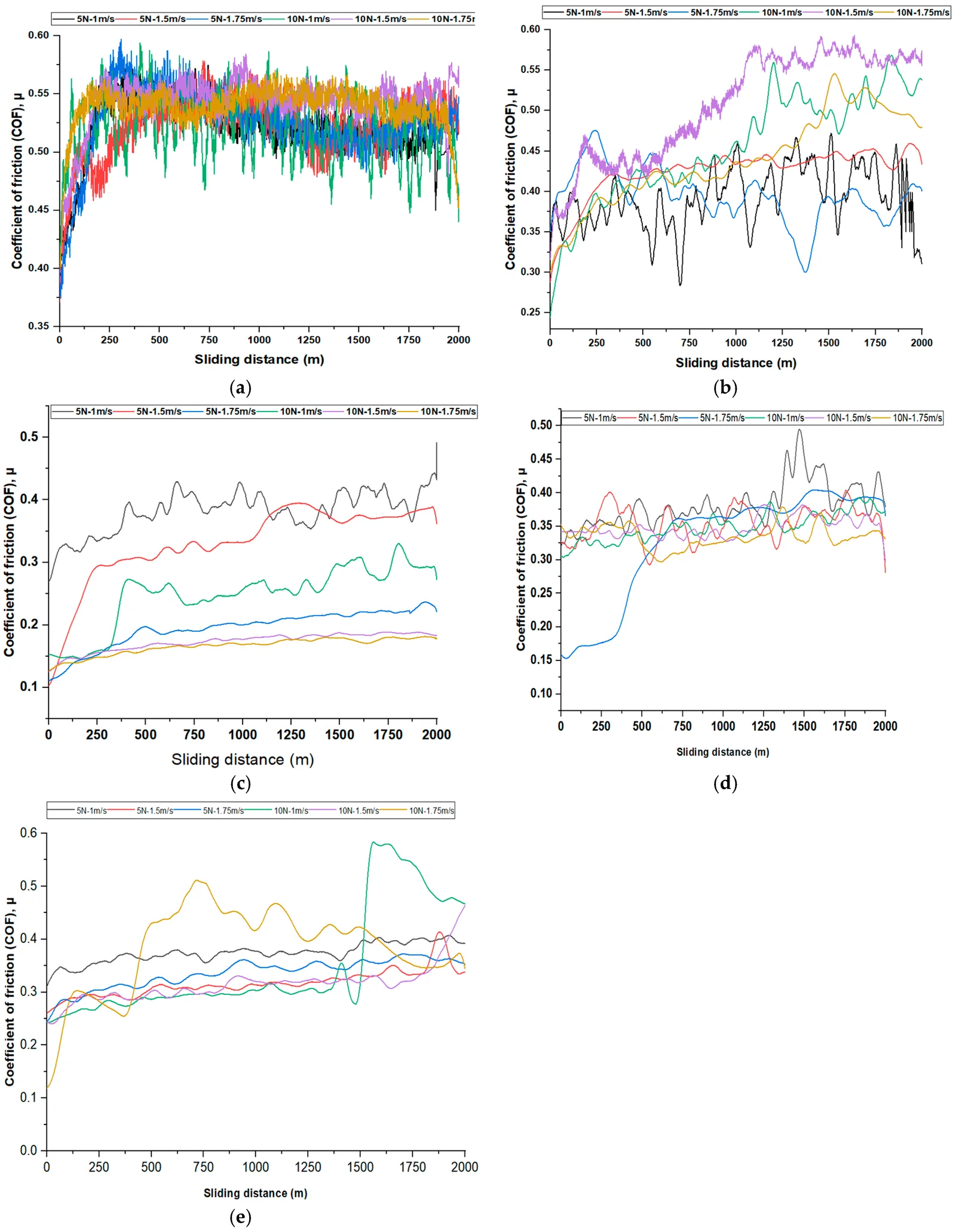
Variation in the instantaneous coefficient of friction (COF) with sliding distance (0–2000 m) under different normal loads and sliding speeds for (**a**) neat epoxy and Te NW–TiB_2_/epoxy composites containing (**b**) 0.25 wt.%, (**c**) 0.50 wt.%, (**d**) 0.75 wt.%, and (**e**) 1.00 wt.% Te NW–TiB_2_. Each curve represents the point-by-point mean of three tests.

Figure 5 shows the instantaneous COF variation of neat epoxy and 0.25%, 0.50%, 0.75%, and 1.00% Te NW–TiB_2_/epoxy samples over a distance of 0–2000 m. Each curve represents the pointwise average of measurements from three experiments conducted under the same load and velocity conditions at the same sliding distance. For all five formulations, intra-experimental variability (SD, CV, and IQR) is reported for each replicate, while inter-experimental reproducibility is calculated separately from the replicate averages. This distinction prevents signal fluctuations along the sliding distance from being conflated with the reproducibility of experiments.

Neat epoxy curves (Figure 5a) initially exhibited a distinct adaptation zone. The COF rose rapidly over the first 200–300 m before approaching a level that fluctuated mostly between 0.52 and 0.56. This initial increase may be associated with the adaptation of surface protrusions, an increase in the actual contact area, the development of adhesive interactions between the epoxy and steel, and the rearrangement of initial wear debris within the contact zone. The curves’ persistence within a similar range thereafter suggests that the contact did not become entirely invariant; rather, a dynamic equilibrium developed between material removal and interface reformation. Previous studies have reported that the thermal conditions resulting from velocity, load, and friction collectively affect the COF in neat epoxy–steel contact [35]. The relatively high COF maintained by neat epoxy over 2000 m demonstrates that the unreinforced matrix retains high shear resistance in contact with the steel counter surface. Zhang et al. reported that the COF of an unreinforced epoxy coating increased from approximately 0.41 at the beginning of sliding to approximately 0.60 at the end of the experiment; microcracks and material rupture were observed on the worn surface [36]. Other studies have likewise reported that the COF of neat epoxy can increase with sliding time or distance under different experimental conditions [37,38]. These literature COF values are cited only for qualitative context, because test configurations, counterface materials, and operating conditions differ from those of the present study.

In the 0.25% composite (Figure 5b), the mean COF ranged from 0.404 to 0.419 under 5 N and from 0.406 to 0.485 under 10 N. Although these values were lower than those of neat epoxy, the COF did not decrease consistently as the sliding speed increased. The observed local increases and decreases, particularly under certain conditions, suggest that the low filler ratio did not stabilize the contact interface equally across all load–velocity conditions. The limited filler content may have caused the effects of hard particles and nanowires within the actual contact area to remain localized.

The 0.50% composite (Figure 5c) exhibited the most pronounced and consistent response to sliding speed among the reinforced formulations. As the speed increased from 1.00 to 1.75 m/s, the overall mean COF decreased from 0.397 ± 0.019 to 0.197 ± 0.005 (50.4%) under 5 N, and from 0.256 ± 0.015 to 0.181 ± 0.027 (29.3%) under 10 N. At the same speeds, increasing the load from 5 N to 10 N reduced the COF by 35.5%, 43.0%, and 8.1%, respectively. The curves approached a lower COF band, particularly after the adaptation section at 1.50 and 1.75 m/s. This decrease may be associated with the shorter interaction time between opposing asperities at higher speeds, as well as changes in the shear response of the near-surface epoxy layer caused by frictional heating [35]. With increasing load, rearrangement of the actual contact area and redistribution of contact pressure may also have contributed to friction force not increasing at the same rate as the normal force. In particular, the low average COF and relatively low SD, CV, and IQR values at 10 N and 1.50 and 1.75 m/s indicate that this formulation offers both a low friction level and temporal stability. However, the effects of speed and load have been reported to depend on the composition of the epoxy system, with some composites exhibiting opposite trends [39,40].

In the 0.75% composite (Figure 5d), the overall mean COF remained within narrow ranges: 0.367–0.382 under 5 N and 0.364–0.375 under 10 N. The lowest COF occurred at 1.50 m/s under both loads, then increased at 1.75 m/s, indicating a nonlinear speed effect. Although the lowest inter-experimental SD (0.014) was observed at 10 N and 1.50 m/s, indicating high repeatability, the CV of 18.85% in the third repetition under the same condition shows that intra-experimental fluctuations can still be substantial. This demonstrates that similar repetition averages do not necessarily indicate consistent stability throughout sliding.

In the 1.00% composite (Figure 5e), the COF did not reach a stable level directly, particularly under 10 N; instead, it showed gradual increases, sudden rises, and subsequent decreases. Under 5 N, the overall average COF decreased from 0.396 ± 0.022 to 0.350 ± 0.032 as speed increased from 1.00 to 1.75 m/s. In contrast, under 10 N, the lowest value, 0.334 ± 0.032, occurred at 1.50 m/s and increased to 0.397 ± 0.029 at 1.75 m/s. Additionally, the high CV values at 10 N and 1.00 and 1.75 m/s indicate that load and speed together may adversely affect friction stability. The sudden fluctuations may be related to the intermittent accumulation and removal of wear residues; however, nanowire agglomeration, insufficient wetting, or transfer-film degradation cannot be established directly from the available COF data.

When Figure 5a–e are considered together, neat epoxy remained in the highest friction band, while the hybrid filler exhibited a concentration-dependent, nonlinear effect. The 0.50% composite showed relatively limited scatter and low COF, particularly at high speeds under a 10 N load. Considering COF, intra-experimental stability, inter-experimental repeatability, and percentage mass loss together, the defensible overall ranking of the four reinforced formulations is 0.50% > 0.75% > 1.00% > 0.25%. This does not mean that the 0.50% formulation yields the lowest COF under every condition; rather, it reflects overall performance across six load–speed conditions. Neat epoxy, tested under identical conditions, serves as the unreinforced reference for both friction and mass loss and is included in the multi-criteria evaluation in Section 4.

To compare the friction behavior of neat epoxy with that of epoxy composites containing different proportions of Te NW–TiB_2_, COF results from three experiments were analyzed. Table 1 reports the average COF for each load and sliding-speed condition across the three replicates, the overall mean, and the inter-experimental standard deviation. In addition, friction-signal variability over the sliding distance in each experiment was assessed using the standard deviation (SD), coefficient of variation (CV), median, and interquartile range (IQR). These statistics were used to evaluate the hybrid filler’s effects on average friction, within-experiment friction stability, and repeatability across experiments.

Neat epoxy served as the baseline comparison group for evaluating the effect of Te NW–TiB_2_ addition on friction behavior. Under the load and velocity conditions studied, the overall mean COF values for neat epoxy ranged from 0.524 to 0.583 (Table 1). The increase in mean COF with sliding velocity, particularly under 10 N, indicates that neat epoxy–steel contact is sensitive to combinations of high load and high velocity. Although mean COF values across the three experiments were similar under some conditions, increases in intra-experimental SD, CV, and IQR showed that the mean friction level and the temporal stability of the friction signal are distinct characteristics. Adding 0.25 wt.% Te NW–TiB_2_ reduced average COF compared with neat epoxy under all load–speed conditions. However, high CV and wide IQR values in some replicates indicated that lower friction did not always correspond to a more stable friction track. The greatest COF reduction was observed in the composite containing 0.50 wt.% Te NW–TiB_2_. As sliding speed increased from 1.00 to 1.75 m/s, the overall average COF decreased from 0.397 to 0.197 under 5 N and from 0.256 to 0.181 under 10 N. This composition produced the lowest overall average COF in five of the six experimental conditions. However, under the 5 N-1.75 m/s condition, the inter-experimental SD was only 0.005, while the CV for R3 was 30.70%. This indicates that average COF values across the three experiments may be highly reproducible even when substantial fluctuations occur within a single experiment. Therefore, performance should not be evaluated solely on the basis of overall average COF.

Although the 0.75% by weight composite did not produce the lowest COF values under all conditions, it exhibited a narrower average COF range across different loads and speeds. This suggests that its average friction behavior is more stable under varying operating conditions. However, high CV values in some repetitions indicate that greater stability in average COF across conditions does not necessarily correspond to temporal stability within each friction curve. Increasing the filler content to 1.00% by weight did not further improve the average COF compared with the 0.50% composition. Moreover, higher inter-experimental SD and intra-experimental CV and IQR values under certain conditions indicate greater variability in friction behavior at higher filler contents. This variability may be related to filler-particle distribution in the contact zone, local particle agglomeration, and irregular transport of third-body material. Because the present friction data do not directly demonstrate these mechanisms, however, these explanations should be regarded as possibilities [35,39,40]. Mean COF and median values describe the central tendency of the friction level, while SD, CV, and IQR provide complementary measures of how stable that level remains throughout the experiment. IQR is particularly useful for assessing the underlying distribution of the COF signal because it is less affected by transient friction peaks. Differences between the mean COF and median suggest that some curves have asymmetric distributions and that transient friction increases can affect the mean. Thus, the indicators presented in Table 1 distinguish average friction performance from intra-experimental fluctuations.

When all compositions were evaluated together, adding Te NW–TiB_2_ reduced the average coefficient of friction (COF) relative to neat epoxy under all six experimental conditions. The 0.50 wt.% composite performed best overall in reducing average friction, while the 0.75 wt.% composite showed more stable behavior across the operating conditions. By contrast, the high CV and IQR values observed for some 0.25 wt.% and 1.00 wt.% compositions indicate that a low average COF does not necessarily imply stable, reproducible friction behavior. These trends may be related to filler–matrix interactions, changes in the actual contact area, and the behavior of third-body material within the contact area [35,36,37,38,39,40].

The three experiments indicate that, among the reinforced formulations studied, the composition containing 0.50% by weight has the lowest average COF. However, given the limited number of replicates and the increased intra-experimental variability observed under certain conditions, it should not be considered the “absolute optimum.” The findings instead suggest that a 0.50% Te NW–TiB_2_ ratio is the most suitable candidate within the experimental range studied.

### 3.4. Quantitative Evaluation of Mass Loss

**Figure 6:**
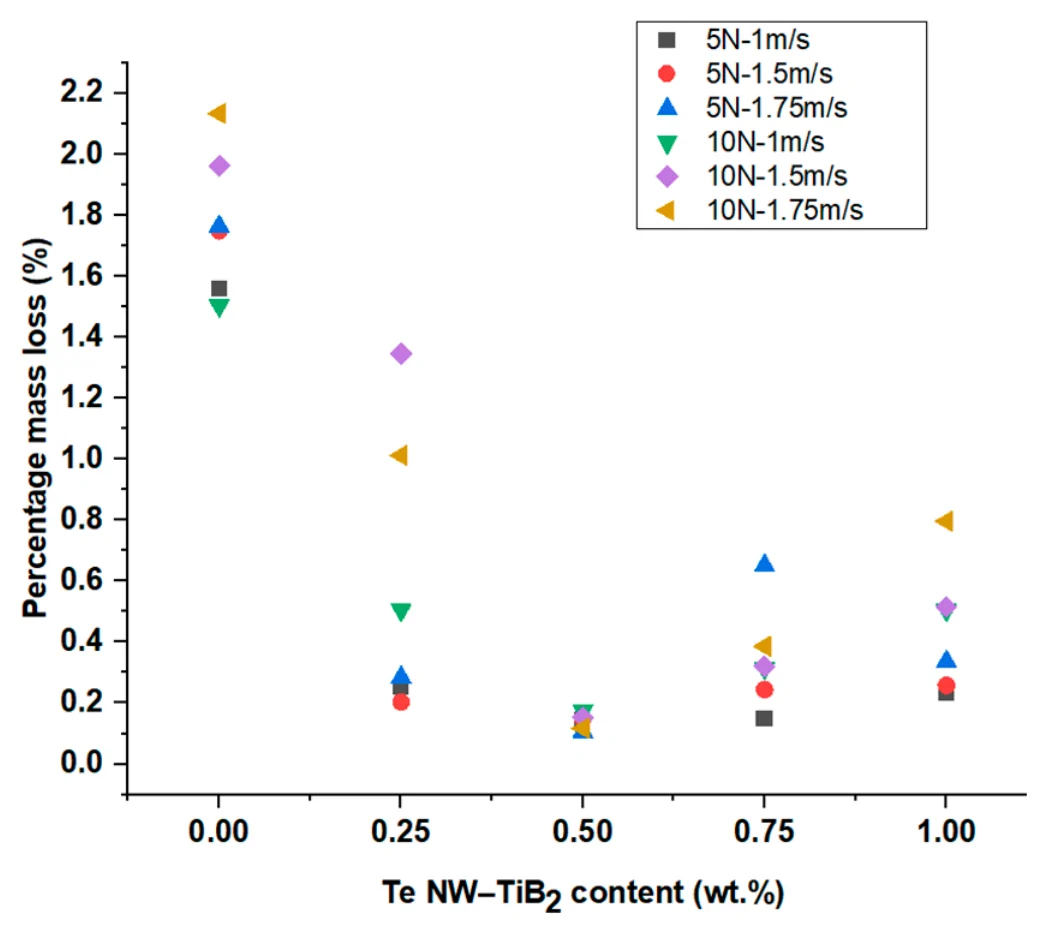
Percentage Mass Loss of Te NW–TiB_2_/Epoxy Composites under Various Normal Loads and Sliding Speeds.

Figure 6 shows the percentage mass loss of neat epoxy (0.0 wt.%) and epoxy samples containing 0.25%, 0.50%, 0.75%, and 1.00% Te NW–TiB_2_ under normal loads of 5 and 10 N and sliding velocities of 1.00, 1.50, and 1.75 m s^−1^. The values shown are percentage mass losses calculated using Equation (2); they are not volumetric or specific wear rates. Neat epoxy exhibited mass losses of approximately 1.5–2.1% under all experimental conditions, significantly higher than those of most reinforced samples. In particular, the increase in material loss at velocities of 1.50 and 1.75 m/s under a 10 N load highlights the unreinforced matrix’s limited resistance to the combined effects of load and velocity. By contrast, the effect of the hybrid filler was nonlinear: the highest filler ratio did not produce the lowest mass loss under every condition.

The six data points for the 0.50% composite were clustered within a narrow, low range, as shown in Figure 6. This pattern becomes more evident when considered alongside the time-dependent friction curves (Figure 5c) and repetition-level statistics. For this composition, the overall mean COF decreased from 0.397 ± 0.019 to 0.197 ± 0.005 under 5 N as velocity increased from 1.00 to 1.75 m/s, and from 0.256 ± 0.015 to 0.181 ± 0.027 under 10 N (Table 1). Moreover, low SD, CV, and IQR values were observed in most repetitions under the 10 N-1.50 m/s and 10 N-1.75 m/s conditions. Thus, the low percentage mass loss in the 0.50% composition was accompanied by a low mean friction level and relatively limited intra-experimental COF scatter.

The results for the 0.75% and 1.00% composites indicate that average COF cannot be linearly correlated with material loss alone. For the 0.75% composite, percentage mass loss was higher under the 5 N-1.75 m/s condition than under the other conditions for the same composition. However, the overall average COF remained at 0.382 ± 0.024 (Figure 6 and Table 1). Similarly, for the 1.00% composite, the high mass loss under the 10 N-1.75 m/s condition coincided with a higher overall average COF of 0.397 ± 0.029, CV values of 20.73–23.17% across repetitions, and IQR values of 0.091–0.095. These findings indicate that certain load–velocity combinations at high filler ratios can increase both material loss and fluctuations in the friction signal.

When Figure 5 and Figure 6 are considered alongside Table 1, the composite containing 0.50% Te NW–TiB_2_ exhibits the most favorable overall tribological performance among the compositions studied, with respect to friction level, friction variability, and percentage mass loss. This finding does not establish 0.50% as a universal or absolute optimum; rather, it identifies it as the best-performing concentration tested under the conditions of this study. The non-monotonic effect of filler concentration in epoxy composites, with more favorable tribological behavior at an intermediate concentration, is also consistent with the trend reported for BNNP-reinforced epoxy systems [41].

### 3.5. Effect of Filler Content and Sliding Conditions on Surface Temperature

**Figure 7:**
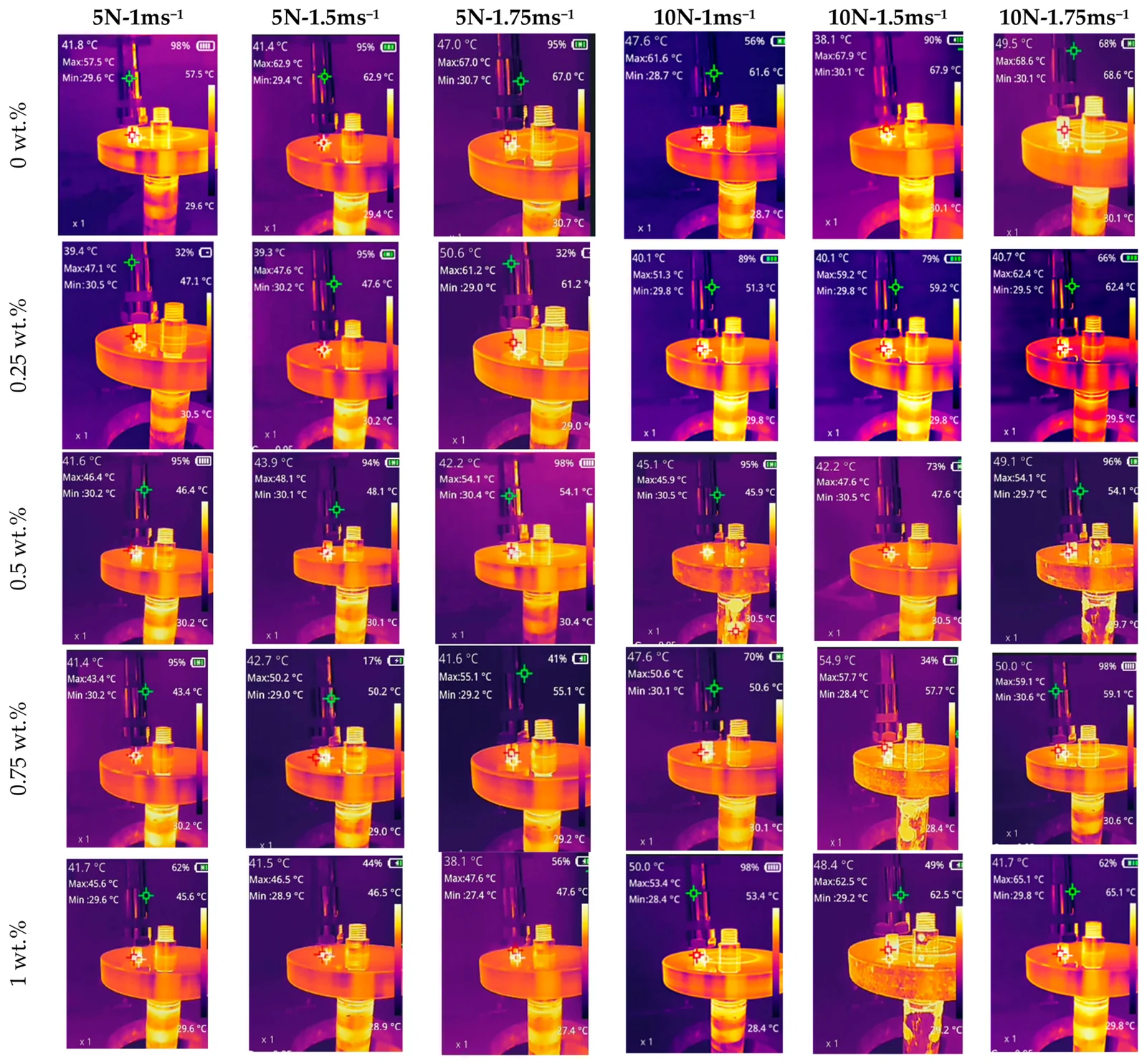
Thermal imaging data illustrating the maximum surface temperatures for Te NW–TiB_2_/epoxy composites, analyzed across varying filler contents, normal loads, and sliding speeds.

In neat epoxy samples (Figure 7, 0 wt.% panel), the maximum surface temperature reached 68.6 °C under the 10 N-1.75 m/s condition, significantly exceeding the 57.5 °C measured under the 5 N-1.00 m/s condition. The higher surface temperature measured at the higher load and sliding velocity is consistent with increased frictional heating under combined load–velocity effects; however, a higher infrared surface temperature is not in itself direct evidence of increased heat generation. This finding is consistent with neat epoxy’s high average COF values (0.525–0.583 under 10 N; Table 1) and its highest percentage mass loss (1.5–2.1%; Figure 6). Thus, high COF, material loss, and surface temperature occurred together under the same conditions in neat epoxy. However, this correlation alone cannot establish whether the temperature increase resulted solely from increased friction or from changes in heat transfer.

**Figure 8:**
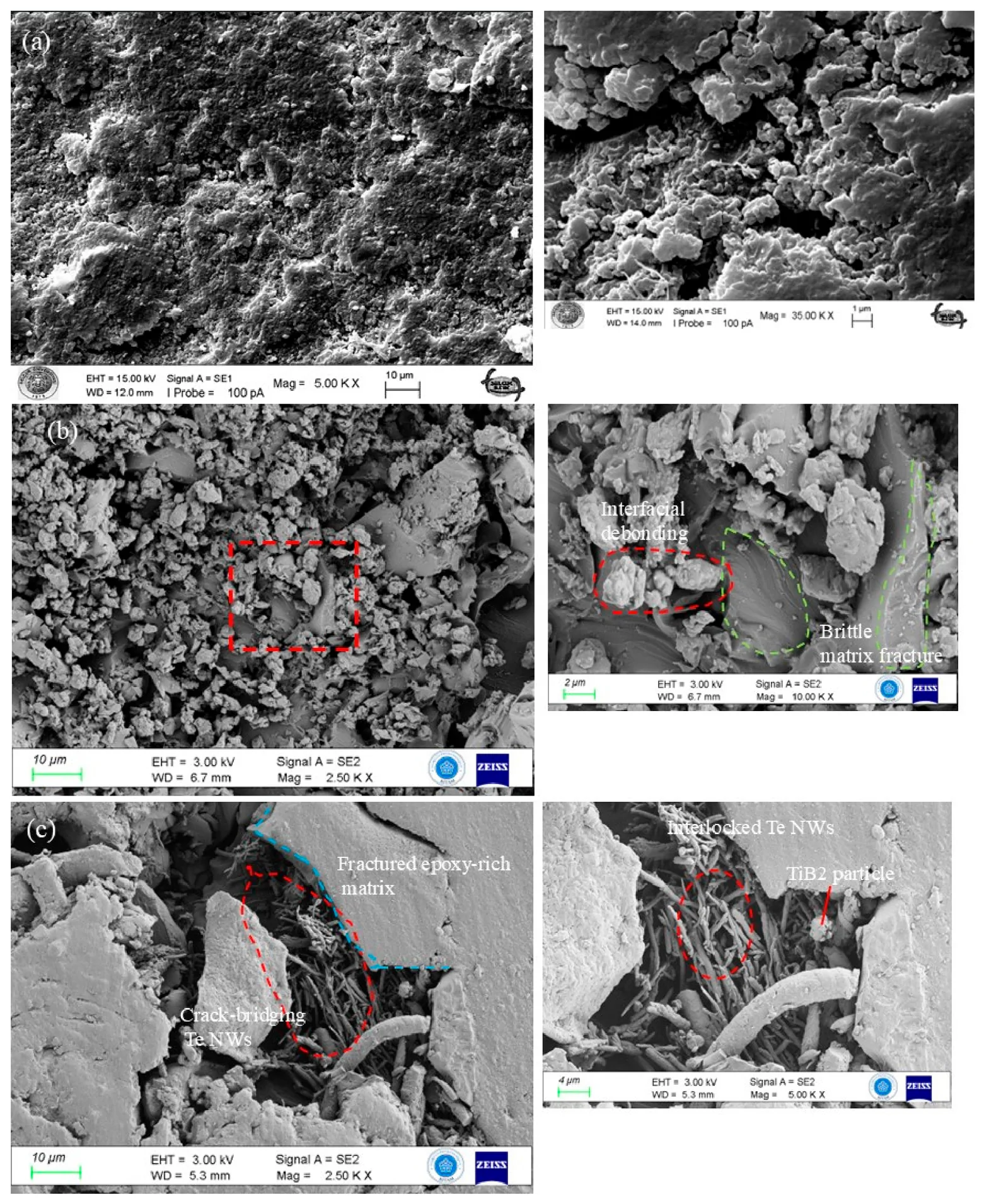

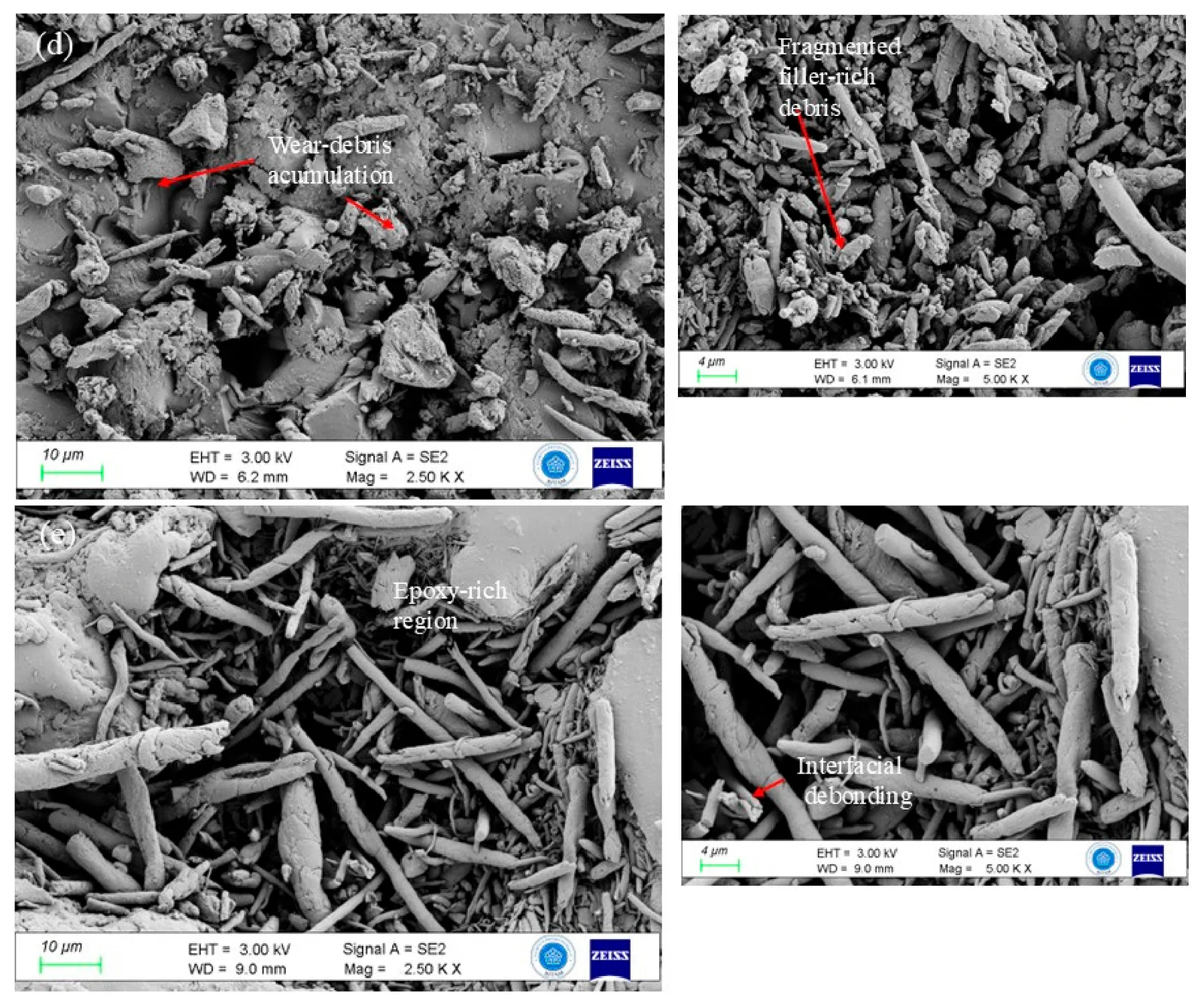
FESEM micrographs of the worn surfaces of Te NW–TiB_2_/epoxy hybrid composites with distinct filler concentrations: (**a**) 0 wt.%, (**b**) 0.25 wt.%, (**c**) 0.50 wt.%, (**d**) 0.75 wt.%, and (**e**) 1.00 wt.%.

In the 0.25% composite (Figure 7, 0.25 wt.% panel), maximum surface temperatures of 61.2, 51.3, and 62.4 °C were measured under 5 N-1.75 m/s, 10 N-1.00 m/s, and 10 N-1.75 m/s, respectively. These values are lower than those for neat epoxy under the same conditions. However, the 0.25% composite exhibited high average COF values (0.406–0.485 under 10 N) and percentage mass losses (approximately 0.51% under 10 N-1.00 m/s and 1.35% under 10 N-1.50 m/s). This suggests that the low filler content reduces surface temperature only to a limited extent and does not provide a consistent improvement in friction and wear behavior. The worn-surface FESEM images in Figure 8b support this interpretation, showing large resin-rich regions, interfacial separation, brittle matrix fracture, and loose residues at 0.25% filler. This morphological degradation suggests that hard TiB_2_ particles may act as abrasive third bodies at the sliding interface [42]. However, this mechanism has not been directly confirmed by the available COF and temperature data.

In the 0.50% composite (Figure 7, 0.5 wt.% panel), the maximum measured surface temperature increased from 45.9 °C to 54.1 °C as velocity increased from 1.00 to 1.75 m/s under a 10 N load. Under the same conditions, the overall mean COF decreased from 0.256 ± 0.015 to 0.181 ± 0.027, while percentage mass loss fell from 0.17% to 0.12% (Table 1 and Figure 6). Most replicates under the 10 N-1.50 and 10 N-1.75 m/s conditions also showed low SD, CV, and IQR values. Although the temperature increased moderately with speed, the maximum surface temperature at 10 N-1.75 m/s (54.1 °C) remained lower than those of neat epoxy (68.6 °C) and the 1.00% composite (65.1 °C) under the same condition. This lower temperature is consistent with the lower mean COF and smaller intra-experimental scatter of the 0.50% composite, and thus with lower frictional heating; however, it cannot be taken as direct evidence of reduced heat generation, nor can it be attributed solely to improved heat conduction, as the composite’s thermal conductivity and thermal diffusivity were not measured independently [43,44].

In the 0.75% composite (Figure 7, 0.75 wt.% panel), the maximum surface temperatures under 10 N were 57.7 °C at 1.50 m/s and 59.1 °C at 1.75 m/s. Under these same conditions, the low CV values (averages of 9.66% and 7.71%) and narrow IQR values (averages of 0.0248 and 0.0367) indicate that the COF distribution remained relatively narrow over the 0–2000 m range (Table 1). These data enable a combined assessment of temperature and friction variability but do not establish a causal heat-transfer mechanism or volumetric nanowire percolation. By contrast, the 0.75% composite showed significantly greater mass loss (0.65%) at 5 N and 1.75 m/s than under the other conditions for the same composition, while the average COF remained 0.382 ± 0.024 (Table 1 and Figure 6).

In the 1.00% composite (Figure 7, 1 wt.% panel), the maximum surface temperature remained between 45.6 and 47.6 °C under a 5 N load, but increased to 65.1 °C under the 10 N-1.75 m/s condition. Under this condition, the overall mean COF was also high (0.397 ± 0.029), with replicate CV values of 20.73–23.17%, IQR values of 0.091–0.095, and a percentage mass loss of approximately 0.80% (Table 1 and Figure 6). These results indicate that certain load–velocity combinations at high filler ratios can increase both material loss and friction-signal fluctuation. The worn-surface FESEM images in Figure 8e show pronounced nanowire entanglement and Te NW–TiB_2_ clustering in this composition, together with filler clusters that appear incompletely embedded in the surrounding matrix. Because worn-surface images alone cannot establish the degree of resin infiltration, this feature is interpreted only as being consistent with locally poor wetting. These morphological observations suggest that filler agglomerates at high loading may act as stress concentrators and promote particle pull-out [45,46].

When Figure 7 is considered as a whole, the measured maximum surface temperatures do not vary monotonically with filler ratio. Although neat epoxy reached the highest temperatures, the 0.50% composite provided the best overall balance, particularly under 10 N, combining a low average COF and low percentage mass loss with a relatively low maximum surface temperature. The 0.75% composite showed a stable friction signal and low temperatures under certain conditions, but mass loss increased for some load–speed combinations. In contrast, the 1.00% composite exhibited friction fluctuations, high mass loss, and elevated surface temperatures, particularly at high loads and speeds. 

However, the temperature assessment presented in this section has two important limitations. First, infrared thermography provided the maximum surface temperature from thermograms recorded at a sliding distance of approximately 1750 m, rather than a continuous temperature–time history over the course of the experiment. Accordingly, we do not use the term “temperature evolution”; instead, we report only the maximum surface temperature at a specific sliding distance [43,44]. Second, we did not measure the thermal conductivity or thermal diffusivity of the composites, nor did we assess heat sharing between the contacting bodies. Therefore, we do not attribute the lower measured surface temperature to a specific percolation network or enhanced heat conduction mechanism; rather, we consider it a finding consistent with reduced friction-induced heat generation and/or altered heat transfer [45]. For the same reason, higher measured surface temperatures, such as those of neat epoxy and the 1.00% composite at 10 N-1.75 m/s, are interpreted as consistent with greater frictional heating and/or less effective heat dissipation, not as direct proof of increased heat generation.

### 3.6. FESEM Analysis of Worn Surfaces and Wear Mechanisms

FESEM micrographs of the worn surfaces (Figure 8) indicate that adding Te NW–TiB_2_ hybrid filler to the epoxy matrix does not linearly improve friction and wear behavior. Instead, it produces surface morphologies that vary significantly with filler ratio. Considered alongside the average friction coefficient, intra-experimental friction-signal variability, inter-experimental repeatability, percentage mass loss, and maximum surface temperature, these morphological differences provide a meaningful overall picture.

The worn surface of the neat epoxy sample exhibits extensive, continuous damage, including prominent cracks, brittle matrix fracture, material spalling, and an irregular wear pattern. No protective layer or consistent traces of third-body material are observed. This morphology suggests that the unreinforced epoxy matrix cannot withstand shear stresses with sufficient load-carrying capacity. The continuous crack network and material spalling are consistent with neat epoxy’s highest average coefficient of friction and greatest percentage mass loss under high-load conditions.

In the composite with a low filler ratio (0.25% Te NW–TiB_2_), the surface morphology is distinctly heterogeneous, with large resin-rich regions coexisting with isolated filler-rich areas. High-magnification images reveal interfacial separation, brittle matrix fracture, particle pull-out cavities, and loosely attached remnants. The nanowires are not uniformly distributed across the surface and form clumps in some areas. This morphology suggests that, at a low filler ratio, the hybrid phase may not form a continuous load-bearing network within the epoxy matrix. Interfacial separation and particle pull-out cavities suggest that high shear stresses are concentrated primarily in the unreinforced epoxy matrix. The detached, hard TiB_2_ particles may act as abrasive third bodies at the sliding interface and intensify micro-cutting and ploughing. This microstructural degradation is consistent with the high average friction coefficient, percentage mass loss, and maximum surface temperature observed under high load in the 0.25% composite. However, this association does not directly demonstrate an abrasive third-body mechanism; it provides only morphological evidence supporting this possibility.

Compared with the other composites studied, the worn surface of the composite containing 0.50% Te NW–TiB_2_ is smoother and shows less pronounced damage. Elongated nanowire structures and thin residue layers are visible in some areas. These features were assessed alongside the low average friction coefficient, low percentage mass loss, and narrow friction distribution measured for this composite. The low standard deviation, coefficient of variation, and interquartile range, particularly under high-load and high-speed conditions, indicate that the friction signal is relatively narrowly distributed along the sliding distance.

Similarly, a smoother surface morphology alone does not demonstrate the formation of a potentially protective transfer film. Therefore, given the available data, the low friction and wear observed in the 0.50% composite may be associated with a worn-surface morphology showing relatively well-distributed nanowires and comparatively mild surface wear features; this association does not by itself establish the underlying mechanism. The worn surface of the composite containing 0.75% Te NW–TiB_2_ showed increased residue formation and localized filler agglomeration. It featured scattered Te-rich flakes, dispersed fine residues, and an irregular third-body morphology. High-magnification images revealed nanowire agglomerates and features suggestive of limited local matrix–filler wetting. The low coefficient of variation and narrow interquartile range measured at high loads and sliding speeds suggest that the friction coefficient remains relatively stable over the sliding distance. This supports the possibility that the 0.75% composite establishes a stable friction regime under certain conditions.

At the highest filler ratio, 1.00% Te NW–TiB_2_, the worn surface morphology was significantly degraded. The worn surface revealed apparent local clustering and entanglement of the nanowires alongside Te NW–TiB_2_ agglomerates. Large agglomerated regions, particle pull-out cavities, and severe third-body abrasion marks predominated. High-magnification images revealed gaps at the matrix–filler boundaries and localized voids; these features may indicate weakened interfacial bonding and locally poor wetting of the filler clusters, although the degree of resin infiltration cannot be determined from worn-surface images alone. These features suggest that nanowire agglomeration may become a dominant damage feature at high filler ratios. Agglomerated filler regions may act as stress concentrators, promoting particle pull-out and interfacial degradation. This may cause the cyclical release of abrasive third bodies, potentially disrupt transfer-film continuity, and intensify third-body abrasive wear. This interpretation is consistent with the high average coefficient of friction, coefficient of variation, interquartile range, percentage mass loss, and maximum surface temperature observed in the 1.00% composite under high-load, high-speed conditions.

Overall, the worn-surface morphologies were consistently associated with the measured tribological performance. At a filler concentration of 0.50%, the surface was smoother and less damaged, and the composite exhibited the lowest average coefficient of friction, lowest percentage mass loss, and a narrow friction distribution. At both lower and higher filler concentrations, the surface morphology became more irregular, consistent with greater friction variability, increased material loss, and rising surface temperature.

## 4. Ranking of Experimental Conditions via TOPSIS, VIKOR, WASPAS, and GRA Methods

The evaluation of tribological performance was not based on a single parameter, such as the mean coefficient of friction (COF). Instead, it was framed as a multi-criteria optimization problem that integrates performance indicators that may compete or conflict. The objective is to find a balanced solution that simultaneously reduces friction resistance, maintains signal stability over time, and minimizes material loss. The decision-making framework presented in this section builds logically on the parametric and descriptive statistical analyses discussed earlier in the paper.

### 4.1. Identification of Alternatives and Evaluation Criteria

The decision-making structure was established at two distinct levels. At the macro level, the alternatives included neat epoxy as an unreinforced reference and four hybrid filler formulations: epoxy composites reinforced with 0.25, 0.50, 0.75, and 1.00 wt.% Te NW–TiB_2_. At the operational level, each formulation was evaluated under six tribological testing conditions, combining two normal loads (5 and 10 N) with three sliding speeds (1.00, 1.50, and 1.75 m/s). This resulted in 30 unique material-operational alternatives (five formulations × six conditions).

To comprehensively evaluate tribological characteristics, five performance metrics were selected: mean coefficient of friction (mean COF), standard deviation (SD), coefficient of variation (CV), interquartile range (IQR), and percentage mass loss. All are cost-type criteria, for which lower values are preferable; thus, the problem is formulated as a minimization problem. The criteria are nevertheless not mutually consistent; for example, neat epoxy has the highest mean COF and mass loss but the lowest dispersion metrics (Section 4.5.2). Because the stability-related metrics (SD, CV, and IQR) are multicollinear, an objective weighting algorithm was used to account for intercriterion correlations and contrast intensity while avoiding redundant calculations. Statistical friction metrics were calculated from the replicate-specific data in Table 1: mean COF is the overall mean across the three tests, whereas SD, CV, and IQR are the averages of their respective values across the three replicates. Percentage mass loss was obtained from the experimental data shown in Figure 6.

### 4.2. Determination of Objective Criteria Weights

To address subjective bias associated with expert preferences, criterion weights were derived objectively from the decision matrix using the CRITIC (Criteria Importance Through Intercriteria Correlation) method [47]. CRITIC assesses each criterion’s contrast intensity, measured by its standard deviation, and its linear conflict with other criteria, measured by correlation coefficients. Criteria providing substantial unique information with minimal redundancy therefore receive greater weights. As an independent validation, the Shannon entropy weighting method [48] was also applied, and the resulting weight vectors were compared. The objective weight vector was calculated once for the full 30 × 5 decision matrix and used consistently in all subsequent ranking processes.

### 4.3. Multi-Criteria Ranking Methodologies

To verify that the best-performing formulation was not simply an artifact of a single computational algorithm, four complementary multi-criteria decision-making (MCDM) methodologies were applied concurrently: TOPSIS, which uses Euclidean distances to the ideal and anti-ideal solutions [48]; VIKOR, which identifies a compromise ranking by balancing maximum group utility (S) and minimum individual regret (R) [49]; WASPAS, which combines the weighted sum model (WSM) and the weighted product model (WPM) [50]; and Grey relational analysis (GRA), which evaluates performance using relational grades relative to a reference sequence [51]. GRA was selected because it is widely used to optimize multi-response experiments in polymer tribology. Because all five criteria are cost-type parameters, the ideal reference targets for all four algorithms were set to the absolute minima.

### 4.4. Robustness and Sensitivity Analysis

The robustness of the resulting rankings was verified in two stages. In the first stage, the level of agreement among the four MCDM methods was quantified with Spearman’s rank correlation coefficient [52]. In the second stage, a sensitivity analysis was performed to examine how the filler rankings respond to changes in criterion weights. For this purpose, six weighting scenarios were considered: CRITIC, Entropy, equal weights, friction-magnitude-biased, stability-biased, and mass-loss-biased. Finally, ranking robustness under worst-case conditions was assessed by examining extreme operational response values within the testing parameter envelope, evaluating model stability under severe contact conditions.

### 4.5. Results of the MCDM Analysis

#### 4.5.1. Evaluation of Objective Criteria Weights

According to the CRITIC method, the criteria were ordered by importance as follows: percentage mass loss, mean COF, CV, IQR, and SD (Table 2). This ordering was largely shaped by the neat epoxy reference. Its mass loss (1.50–2.13%) was roughly one order of magnitude higher than that of most hybrid composites (0.11–1.35%), which markedly increased the contrast intensity of the mass-loss criterion. As a result, percentage mass loss obtained the highest CRITIC weight (0.295). Within the group of statistical stability metrics, CV was assigned the largest weight (0.202), owing to its higher contrast and the non-redundant information it provides. SD (0.148) and IQR (0.151), in contrast, received lower weights, reflecting their stronger correlation with the other stability metrics. In the Shannon Entropy method, mean COF emerged as the most influential criterion (0.276), with percentage mass loss ranking second (0.211). Although the methods ranked the leading criteria differently, both assigned the two highest weights to mean COF and percentage mass loss, which together accounted for 0.500 of the CRITIC weight and 0.487 of the Entropy weight. This confirms that friction magnitude and material loss dominated the decision problem, while the three stability metrics shared the remaining weight. This agreement indicates that the two objective weighting schemes give a consistent picture of the decision problem.

#### 4.5.2. Identification of the Best-Performing Filler Content

When all operating conditions were considered together in the integrated decision matrix, the 0.50 wt.% formulation obtained the best aggregate performance in each of the four MCDM methods (TOPSIS = 0.815, VIKOR Q = 0.248, WASPAS = 0.597, and GRA = 0.777). The 0.75 wt.% formulation ranked second in all cases (Table 3). The gap between these two formulations was narrow in TOPSIS (0.815 vs. 0.802) and VIKOR (0.248 vs. 0.289) but wider in WASPAS (0.597 vs. 0.440) and GRA (0.777 vs. 0.701).

The only disagreement among the methods concerned the neat epoxy reference. TOPSIS and VIKOR ranked it fifth, whereas WASPAS and GRA ranked it third, above the 1.00 and 0.25 wt.% composites. This divergence arises from the different normalization and aggregation procedures of the methods. The neat epoxy exhibited the highest mean COF and mass loss, yet the lowest average SD, CV, and IQR. The ratio-based WASPAS and the grey relational coefficients of GRA penalize such a profile less severely than the distance-based TOPSIS and the regret-based VIKOR.

A worst-case robustness ranking was also performed, in which the least favorable value of each criterion across the six operating conditions was used. Under this scenario, the 0.50 wt.% composite ranked first according to TOPSIS (0.783) and WASPAS (0.788). VIKOR and GRA, however, slightly favored the 0.75 wt.% composite (e.g., GRA: 0.802 vs. 0.784). Nevertheless, the 0.50 and 0.75 wt.% composites ranked in the top two under worst-case conditions across all four methods, indicating that their superior tribological performance was sustained even under the most demanding conditions.

#### 4.5.3. Regime-Dependent Frictional Behavior

**Figure 9:**
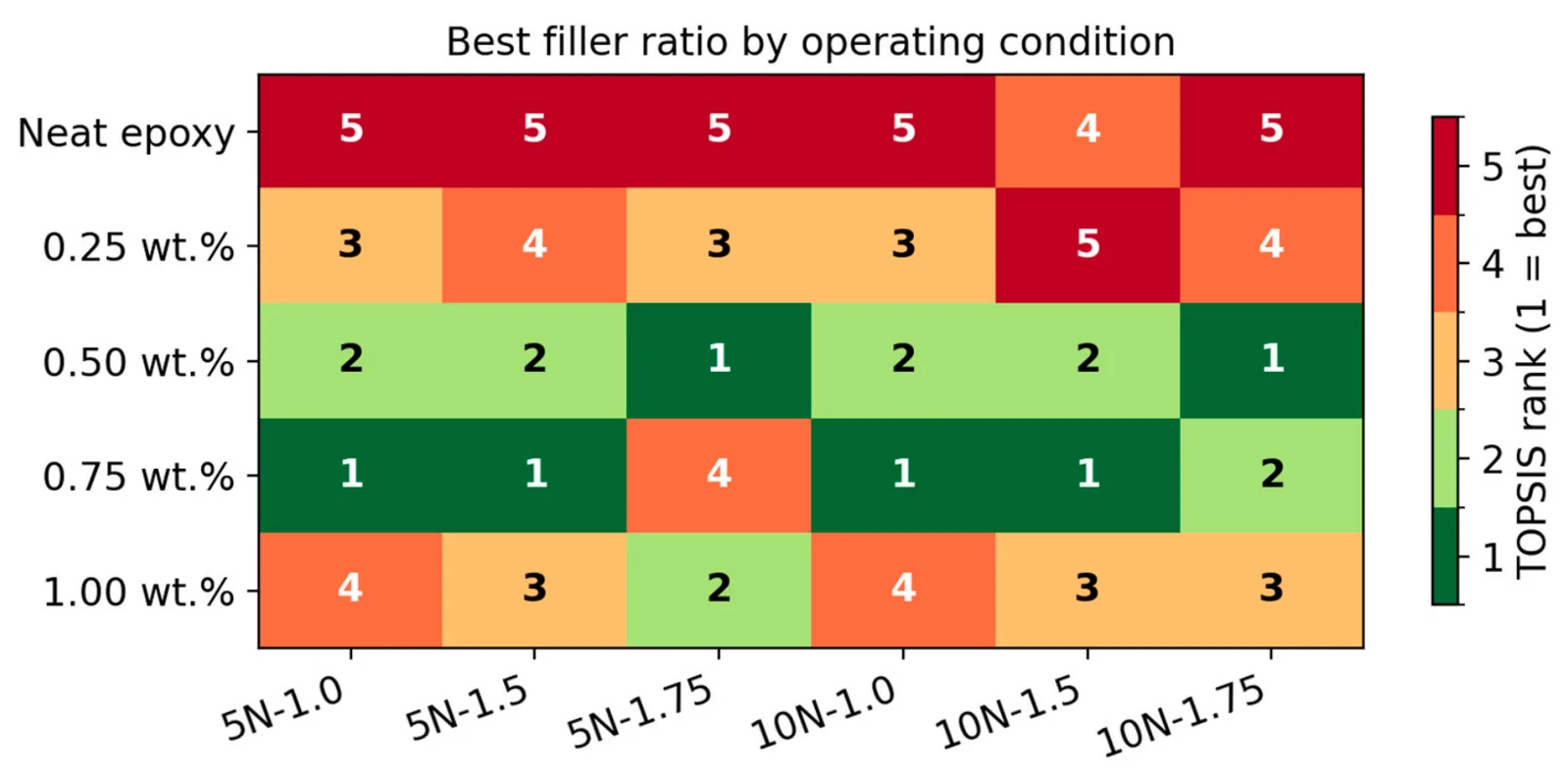
TOPSIS ranking of neat and hybrid filler loadings for each distinct operating condition, where a rank of 1 indicates the best performance.

When the formulations were evaluated separately under each operating condition, the top-performing formulation varied systematically across the test regimes (Figure 9). According to TOPSIS, the 0.50 wt.% composite ranked first at 5 N-1.75 m/s and 10 N-1.75 m/s and second under the other four conditions, never falling below second place. The 0.75 wt.% composite ranked first at 5 N-1.00 m/s, 5 N-1.50 m/s, 10 N-1.00 m/s, and 10 N-1.50 m/s, reflecting its low dispersion metrics (e.g., CV = 9.66% and IQR = 0.025 at 10 N-1.50 m/s). However, it fell to fourth place at 5 N-1.75 m/s, where its mass loss increased to 0.65%. Neat epoxy ranked last under five of the six conditions and fourth at 10 N-1.50 m/s, confirming the benefits of hybrid reinforcement across the full test envelope. The leading formulation for each condition depended on the method: VIKOR matched the TOPSIS selections, GRA selected 0.50 wt.% in four conditions (5 N-1.75 m/s, 10 N-1.00 m/s, 10 N-1.50 m/s, and 10 N-1.75 m/s), and WASPAS selected 0.50 wt.% in all six conditions. This pattern indicates that 0.50 wt.% is the most consistent choice, particularly at the high speed of 1.75 m/s, where all four methods favor it unanimously. By contrast, 0.75 wt.% is competitive at lower sliding speeds, where frictional stability carries greater weight in the evaluation. These findings support the central argument of this work regarding interactions among load, speed, and filler content.

#### 4.5.4. Cross-Method Consistency and Weight Sensitivity Analysis

Across the five formulations, Spearman’s rank correlation coefficient was 1.00 between TOPSIS and VIKOR and between WASPAS and GRA, while it was 0.70 for all other method pairs. This difference was attributable solely to the position of the neat epoxy reference. Considering only the four hybrid formulations, the coefficient was 1.00 for every method pair, indicating that their preference order was independent of the MCDM algorithm. For the 30 individual material-condition alternatives, Spearman’s coefficients ranged from 0.74 to 0.98, indicating strong agreement among methods.

**Figure 10:**
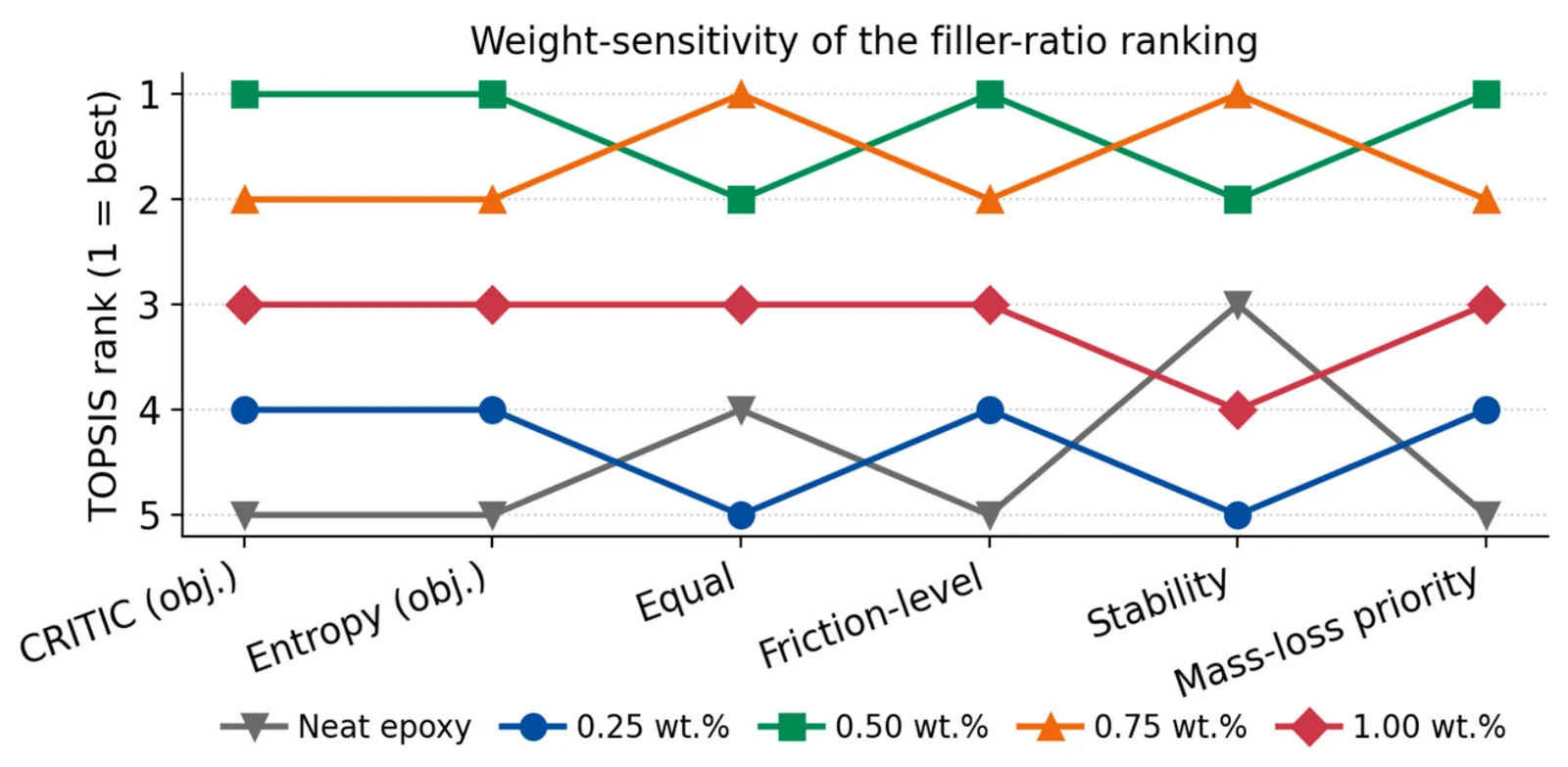
Sensitivity of the TOPSIS ranking of neat epoxy and composite filler loadings across various weighting scenarios. The 0.50 and 0.75 wt.% formulations occupy the top two positions in all cases, with 0.50 wt.% ranked first in four of the six scenarios.

The sensitivity analysis considered six weighting scenarios: CRITIC, Shannon Entropy, equal weights, friction-magnitude-biased, stability-biased, and mass-loss-biased. The 0.50 wt.% formulation held the first position in four of these scenarios. Only under the equal-weight and stability-biased scenarios did it drop to second place, with the 0.75 wt.% composite taking the lead (Figure 10). The 0.50 wt.% and 0.75 wt.% formulations occupied the top two positions in every scenario. When 50% of the weight was assigned to percentage mass loss, the 0.50 wt.% formulation ranked first under all four MCDM methods. The neat epoxy ranked last in four of the six scenarios and rose to third place only under stability-biased weighting, owing to its low friction dispersion. These robustness tests show that the selection of 0.50 wt.% as the best hybrid formulation is robust across computational methods and objective, friction-oriented, and mass-loss-oriented weighting preferences. The 0.75 wt.% formulation is a close alternative when frictional stability is prioritized.

## 5. Conclusions

This study systematically examined how incorporating one-dimensional Te nanowires and TiB_2_ particles as hybrid fillers in an epoxy matrix affects tribological behavior under dry-sliding conditions. It evaluated the effects of filler ratio, normal load, and sliding speed on average coefficient of friction, friction stability, percentage mass loss, and maximum surface temperature within a multi-criteria decision framework.

The original contribution of this study is to demonstrate that the Te NW–TiB_2_ hybrid filler nonlinearly affects the friction and wear performance of the epoxy matrix. It also underscores the need to evaluate this effect not only through the average coefficient of friction but also by considering friction-signal variability within experiments, repeatability across experiments, mass loss, and surface temperature. These findings suggest that, when developing epoxy-based tribological materials, the hybrid filler ratio should be optimized through a multi-criteria balance rather than on the basis of a single parameter.

1. The effect of Te NW–TiB_2_ hybrid filler on the epoxy matrix’s tribological performance is nonlinear: low, medium, and high filler ratios produce significantly different friction and wear regimes. Among the four reinforced formulations evaluated, the best overall balance was achieved at a Te NW–TiB_2_ loading of 0.50 wt.%. This formulation showed the lowest average friction coefficient in five of the six test conditions, the best between-test repeatability, and the lowest percentage mass loss, whereas the 0.75 wt.% formulation showed lower within-test variability of the friction signal under most conditions.

2. The composite containing 0.50 wt.% Te NW–TiB_2_ exhibited the lowest average coefficient of friction and the lowest percentage mass loss, together with a narrow friction distribution, particularly under high-load and high-speed conditions (10 N-1.50 m/s and 10 N-1.75 m/s). At 10 N-1.50 m/s, the overall mean COF was 0.200 ± 0.032, with within-test SD values of 0.0141–0.0519, a CV of 7.96–22.08%, an IQR of 0.019–0.059, and a percentage mass loss of approximately 0.15%. At 10 N-1.75 m/s, the overall mean COF was lowest, at 0.181 ± 0.027, with SD values of 0.0130–0.029, a CV of 7.83–13.61%, an IQR of 0.017–0.027, and a percentage mass loss of 0.12%. The low SD, CV, and IQR values under these conditions indicate a relatively stable friction signal throughout the sliding distance.

3. Neat epoxy exhibited the highest average coefficient of friction, percentage mass loss, and maximum surface temperature across all tested load–speed conditions. This suggests that the unreinforced epoxy matrix lacks sufficient load-carrying capacity under combined loading and speed effects; its higher measured surface temperatures are consistent with greater frictional heating, although heat generation and heat transfer could not be separated with the present measurements. Adding the Te NW–TiB_2_ hybrid filler reduced the average coefficient of friction and mass loss compared with neat epoxy; however, the extent of improvement varied significantly with the filler ratio.

4. Although the composite containing 0.25% Te NW–TiB_2_ had a lower average coefficient of friction than neat epoxy, it exhibited substantial friction variability (CV up to 26.87%; IQR up to 0.196) and high mass loss (0.51–1.35%), particularly under a 10 N load. FESEM images of the worn surfaces show large resin-rich regions, interfacial separation, particle pull-out cavities, and loose debris. This morphological degradation suggests that, at a low filler ratio, the hybrid phase cannot form a continuous load-bearing network, while the hard TiB_2_ particles may act as abrasive third bodies.

5. The composite containing 0.75% Te NW–TiB_2_ exhibited a relatively narrow range of average friction coefficients (0.364–0.382), similar to the 0.50% composition. Under certain conditions, particularly at 10 N-1.50 m/s and 10 N-1.75 m/s, it maintained a stable friction regime, with mean coefficients of variation (CVs) of 9.66% and 7.71% and mean interquartile ranges (IQRs) of 0.0248 and 0.0367, respectively. However, the percentage mass loss increased significantly, reaching 0.65%. This was especially evident under low-load, high-speed conditions (5 N-1.75 m/s), where the mean coefficient of friction (COF) remained at 0.382 ± 0.024. These findings indicate that average friction coefficient and material loss cannot be correlated linearly or considered in isolation. Thus, a 0.75% filler ratio stabilizes the friction signal but does not preserve wear resistance to the same extent.

6. The composite containing 1.00% Te NW–TiB_2_ exhibited reduced tribological stability at the highest filler ratio examined. Under the 10 N-1.75 m/s condition, it showed a high average friction coefficient, coefficient of variation, interquartile range, percentage mass loss, and maximum surface temperature. FESEM images of the worn surface showed Te NW–TiB_2_ agglomerates, nanowire entanglement, and gaps at matrix–filler boundaries, features consistent with locally poor wetting. These features may be associated with the elevated mass loss and friction fluctuations observed at this loading, although worn-surface images alone cannot establish the underlying mechanism.

7. FESEM images of the worn surface at a filler ratio of 0.50% showed a smoother morphology with less pronounced damage. This is consistent with the composite’s low average coefficient of friction, low percentage mass loss, and narrow friction distribution.

8. Multi-criteria decision-making analyses using TOPSIS, VIKOR, WASPAS, and GRA, supported by the CRITIC and Shannon entropy weighting methods, were applied to the decision matrix, with neat epoxy serving as the reference. This yielded a ranking of composites containing four different filler percentages. The ranking reflects not only the average coefficient of friction but also an integrated assessment of competing performance indicators, including intra-experimental variability in the friction signal (SD, CV, and IQR) and percentage mass loss. The only difference among the methods was the position of neat epoxy: it ranked last in TOPSIS and VIKOR, and third in WASPAS and GRA. The 0.50% formulation ranked first in four of the six weighting scenarios but was outperformed by the 0.75% formulation under equal-weight and stability-weighting scenarios. The 0.50% and 0.75% formulations occupied the top two positions across all weighting scenarios and worst-case evaluations. This supports the conclusion that the 0.50% Te NW–TiB_2_ formulation is the most suitable candidate within the experimental range examined, with the 0.75% formulation as a close alternative.

## Figures and Tables

**Table 1:** Replicate-specific friction statistics and between-test reproducibility of the coefficient of friction (COF) for neat epoxy and Te NW–TiB_2_/epoxy composites containing 0.25, 0.50, 0.75, and 1.00 wt.% hybrid filler across the full 0–2000 m sliding interval, under different normal loads and sliding speeds (n = 3 tests per condition). Note: Mean COF values are reported as R1/R2/R3, followed by the overall mean ± between-test SD. Within-test SD, CV, median, and IQR values are reported in the order R1/R2/R3.

	Load (N)	Sliding Speed (m/s)	Mean COF: Overall Mean ± Between-Test SD	SD	CV (%)	Median	IQR
Neat epoxy	5	1	0.534/0.509/0.531: 0.525 ± 0.0137	0.0108/0.0509/0.0313	2.02/10.00/5.89	0.514/0.522/0.525	0.011/0.050/0.036
	5	1.5	0.497/0.556/0.562: 0.538 ± 0.0360	0.0487/0.0556/0.0522	9.79/9.84/9.28	0.491/0.565/0.526	0.018/0.060/0.0340
	5	1.75	0.532/0.523/0.516: 0.524 ± 0.0080	0.0240/0.0512/0.0322	4.51/9.79/6.24	0.533/0.529/0.514	0.033/0.048/0.031
	10	1	0.544/0.486/0.545: 0.525 ± 0.0338	0.0174/0.0404/0.0747	3.20/8.31/13.71	0.548/0.495/0.515	0.020/0.029/0.113
	10	1.5	0.514/0.572/0.583: 0.556 ± 0.0371	0.0182/0.0417/0.0217	3.54/7.29/3.72	0.515/0.580/0.546	0.0230/0.034/0.021
	10	1.75	0.597/0.585/0.566: 0.583 ± 0.0156	0.0211/0.0478/0.0285	3.53/8.17/5.04	0.561/0.553/0.541	0.0150/0.032/0.019
0.25 wt.% Te NW–TiB_2_	5	1	0.394/0.386/0.432: 0.404 ± 0.025	0.035/0.034/0.035	9.11/8.86/8.125	0.392/0.382/0.412	0.052/0.058/0.063
	5	1.5	0.457/0.404/0.396: 0.419 ± 0.033	0.065/0.128/0.103	14.44/31.68/26.01	0.528/0.418/0.432	0.121/0.193/0.203
	5	1.75	0.422/0.441/0.374: 0.412 ± 0.035	0.036/0.039/0.105	8.46/8.84/28.07	0.433/0.442/0.450	0.022/0.038/0.183
	10	1	0.461/0.458/0.448: 0.456 ± 0.007	0.059/0.063/0.079	12.96/13.86/17.63	0.466/0.486/0.562	0.087/0.085/0.189
	10	1.5	0.492/0.512/0.451: 0.485 ± 0.031	0.041/0.104/0.061	8.39/20.31/13.67	0.491/0.478/0.442	0.038/0.093/0.097
	10	1.75	0.438/0.367/0.413: 0.406 ± 0.036	0.056/0.093/0.111	12.77/25.41/26.87	0.431/0.353/0.578	0.079/0.135/0.196
0.5 wt.% Te NW–TiB_2_	5	1	0.382/0.390/0.418: 0.397 ± 0.019	0.0330/0.038/0.041	8.63/9.74/9.80	0.387/0.395/0.37	0.041/0.048/0.086
	5	1.5	0.331/0.364/0.359: 0.351 ± 0.018	0.0678/0.073/0.0389	20.48/20.05/10.83	0.333/0.371/0.393	0.068/0.078/0.061
	5	1.75	0.195/0.193/0.213: 0.197 ± 0.005	0.0290/0.032/0.0654	14.87/16.58/30.7	0.202/0.2014/0.264	0.029/0.0321/0.091
	10	1	0.248/0.247/0.273: 0.256 ± 0.015	0.0481/0.058/0.0857	19.40/23.48/31.39	0.257/0.302/0.295	0.036/0.069/0.064
	10	1.5	0.173/0.192/0.235: 0.200 ± 0.032	0.0141/0.0153/0.0519	8.15/7.96/22.08	0.178/0.189/0.203	0.019/0.056/0.059
	10	1.75	0.166/0.165/0.213: 0.181 ± 0.027	0.0130/0.0142/0.029	7.83/8.60/13.61	0.169/0.173/0.197	0.017/0.023/0.027
0.75 wt.% Te NW–TiB_2_	5	1	0.381/0.359/0.391: 0.377 ± 0.016	0.0326/0.0349/0.0401	8.55/9.70/10.25	0.387/0.353/0.356	0.0406/0.0370/0.0451
	5	1.5	0.329/0.400/0.372: 0.367 ± 0.036	0.0606/0.0335/0.0549	18.37/8.38/14.75	0.332/0.402/0.420	0.0679/0.0471/0.0672
	5	1.75	0.354/0.396/0.395: 0.382 ± 0.024	0.0249/0.0653/0.0492	7.04/16.48/12.45	0.357/0.442/0.379	0.0366/0.0782/0.1040
	10	1	0.349/0.408/0.368: 0.375 ± 0.030	0.0206/0.0488/0.0891	5.90/11.96/24.21	0.347/0.398/0.374	0.0302/0.0447/0.0890
	10	1.5	0.348/0.375/0.368: 0.364 ± 0.014	0.0145/0.022/0.0694	4.16/5.96/18.85	0.344/0.372/0.356	0.0178/0.0217/0.0349
	10	1.75	0.334/0.405/0.373: 0.371 ± 0.036	0.0164/0.0232/0.0465	4.92/5.74/12.46	0.333/0.403/0.362	0.0193/0.0278/0.0631
1 wt.% Te NW–TiB_2_	5	1	0.375/0.395/0.419: 0.396 ± 0.022	0.1018/0.1049/0.1321	27.14/26.58/31.52	0.315/0.444/0.395	0.0251/0.1329/0.1094
	5	1.5	0.317/0.412/0.381: 0.370 ± 0.048	0.0653/0.0556/0.0953	20.59/13.49/25.01	0.337/0.384/0.433	0.0265/0.1021/0.1176
	5	1.75	0.336/0.387/0.328: 0.350 ± 0.032	0.027/0.0859/0.0792	8.03/22.21/24.14	0.344/0.409/0.335	0.041/0.0526/0.0379
	10	1	0.347/0.352/0.428: 0.376 ± 0.045	0.103/0.1047/0.1395	29.64/29.73/32.59	0.297/0.299/0.387	0.067/0.1772/0.1289
	10	1.5	0.314/0.318/0.371: 0.334 ± 0.032	0.034/0.0441/0.0582	10.81/13.85/15.68	0.317/0.315/0.328	0.027/0.0310/0.0305
	10	1.75	0.383/0.378/0.431: 0.397 ± 0.029	0.079/0.0875/0.0989	20.73/23.17/22.94	0.407/0.402/0.417	0.091/0.0921/0.0945

**Table 2:** Decision criteria, operational directions, and objective weight distributions derived from the 30 × 5 decision matrix.

Criteria	Type	CRITIC Weight	Entropy Weight
Mean COF	Cost	0.205	0.276
SD	Cost	0.148	0.193
CV	Cost	0.202	0.193
IQR	Cost	0.151	0.127
Mass loss	Cost	0.295	0.211

**Table 3:** The collective ranking of the neat epoxy and composite filler loadings, averaged across the conditions, is presented. Higher scores indicate superior performance for TOPSIS, WASPAS, and GRA, whereas lower Q values indicate superior performance for VIKOR.

Filler Loading	TOPSIS	Rank	VIKOR (Q)	Rank	WASPAS	Rank	GRA	Rank
Neat epoxy	0.456	5	0.787	5	0.352	3	0.602	3
0.25 wt.%	0.618	4	0.555	4	0.310	5	0.567	5
0.50 wt.%	0.815	1	0.248	1	0.597	1	0.777	1
0.75 wt.%	0.802	2	0.289	2	0.440	2	0.701	2
1.00 wt.%	0.651	3	0.533	3	0.317	4	0.582	4

## Data Availability

The original contributions presented in this study are included in this article and its Supplementary Materials (Table S1). Further inquiries should be directed to the corresponding author.

## Supplementary Materials

The following supporting information can be downloaded at: https://www.mdpi.com/article/10.3390/polym18192380/s1, Table S1: Replicate-level specimen masses before (m_i_) and after (m_f_) sliding, absolute mass loss (Δm = m_i_ − m_f_), and percentage mass loss [(m_i_ − m_f_)/m_i_ × 100%] for neat epoxy and Te NW–TiB_2_/epoxy composites under all load–speed conditions (sliding distance 2000 m; n = 3 tests per condition). The mean values correspond to the data shown in Figure 6 and used in the MCDM decision matrix.
