# Supplementary material for: Friction Stability and Wear Behavior of Te NW–TiB2/Epoxy Composites
Source: Polymers (Basel). 2026 Sep 29;18(19):2380. doi: 10.3390/polym18192380 (PMC13667057; doi:10.3390/polym18192380)
Supplement: Supplementary file 1 [file polymers-18-02380-s001.zip › polymers-4579551-supplementary.pdf]

Supplementary Materials

Table S1. Replicate-level specimen masses before (m_i) and after (m_f) sliding, absolute mass loss ($\Delta m = m_i - m_f$), and percentage mass loss [$(m_i - m_f)/m_i \times 100\%$] for neat epoxy and Te NW–TiB_2/epoxy composites under all load–speed conditions (sliding distance 2000 m; n = 3 tests per condition). The mean values correspond to the data shown in Figure 6 and used in the MCDM decision matrix.

Formulation	Load (N)	Speed (m/s)	Rep.	m_i (g)	m_f (g)	Δm (mg)	Mean ± SD: Δm (mg) / mass loss (%)
Neat epoxy	5	1.00	R1	1.8054	1.7771	28.3	27.7 ± 2.3 / 1.549 ± 0.106
			R2	1.7571	1.7319	25.2	
			R3	1.8064	1.7767	29.7	
Neat epoxy	5	1.50	R1	1.8018	1.7703	31.5	31.2 ± 1.6 / 1.749 ± 0.074
			R2	1.7938	1.7611	32.7	
			R3	1.7611	1.7316	29.5	
Neat epoxy	5	1.75	R1	1.8156	1.7841	31.5	32.0 ± 2.9 / 1.765 ± 0.134
			R2	1.7841	1.7547	29.4	
			R3	1.8362	1.8011	35.1	
Neat epoxy	10	1.00	R1	1.7895	1.7661	23.4	26.6 ± 3.3 / 1.504 ± 0.193
			R2	1.7661	1.7362	29.9	
			R3	1.7537	1.7272	26.5	
Neat epoxy	10	1.50	R1	1.7365	1.7038	32.7	34.2 ± 2.5 / 1.963 ± 0.187
			R2	1.7038	1.6667	37.1	
			R3	1.7983	1.7654	32.9	
Neat epoxy	10	1.75	R1	1.7694	1.7338	35.6	37.7 ± 1.9 / 2.134 ± 0.131
			R2	1.7338	1.6944	39.4	
			R3	1.8032	1.7650	38.2	
0.25 wt.%	5	1.00	R1	1.6226	1.6189	3.7	4.4 ± 0.7 / 0.253 ± 0.034
			R2	1.7503	1.7452	5.1	
			R3	1.8021	1.7978	4.3	
0.25 wt.%	5	1.50	R1	1.5099	1.5072	2.7	3.2 ± 0.6 / 0.204 ± 0.033
			R2	1.5763	1.5725	3.8	
			R3	1.6734	1.6702	3.2	
0.25 wt.%	5	1.75	R1	1.5090	1.5055	3.5	4.5 ± 1.1 / 0.285 ± 0.062
			R2	1.5938	1.5895	4.3	
			R3	1.5894	1.5838	5.6	
0.25 wt.%	10	1.00	R1	1.6189	1.6111	7.8	8.2 ± 0.5 / 0.505 ± 0.035
			R2	1.6111	1.6023	8.8	
			R3	1.6592	1.6511	8.1	
0.25 wt.%	10	1.50	R1	1.6018	1.5786	23.2	22.4 ± 1.9 / 1.346 ± 0.135
			R2	1.6923	1.6721	20.2	
			R3	1.7031	1.6793	23.8	
0.25 wt.%	10	1.75	R1	1.5396	1.5229	16.7	16.6 ± 1.3 / 1.013 ± 0.092
			R2	1.6832	1.6679	15.3	
			R3	1.7036	1.6858	17.8	
0.50 wt.%	5	1.00	R1	1.7516	1.7485	3.1	2.5 ± 0.7 / 0.142 ± 0.042
			R2	1.7485	1.7458	2.7	
			R3	1.7823	1.7806	1.7	
0.50 wt.%	5	1.50	R1	1.7432	1.7412	2.0	2.7 ± 0.6 / 0.150 ± 0.033
			R2	1.7843	1.7811	3.2	
			R3	1.7831	1.7803	2.8	

Formulation	Load (N)	Speed (m/s)	Rep.	m_i (g)	m_f (g)	Δm (mg)	Mean ± SD: Δm (mg) / mass loss (%)
0.50 wt.%	5	1.75	R1	1.6530	1.6506	2.4	1.8 ± 0.5 / 0.106 ± 0.035
			R2	1.7497	1.7483	1.4	
			R3	1.7483	1.7467	1.6	
0.50 wt.%	10	1.00	R1	1.6540	1.6503	3.7	2.9 ± 0.7 / 0.174 ± 0.043
			R2	1.6503	1.6479	2.4	
			R3	1.7493	1.7466	2.7	
0.50 wt.%	10	1.50	R1	1.7430	1.7410	2.0	2.6 ± 0.7 / 0.153 ± 0.040
			R2	1.7410	1.7384	2.6	
			R3	1.7107	1.7074	3.34	
0.50 wt.%	10	1.75	R1	1.6523	1.6507	1.6	2.0 ± 0.4 / 0.117 ± 0.021
			R2	1.6507	1.6488	1.9	
			R3	1.7183	1.7159	2.4	
0.75 wt.%	5	1.00	R1	1.4538	1.4508	3.0	3.0 ± 0.8 / 0.190 ± 0.047
			R2	1.6842	1.6804	3.8	
			R3	1.6804	1.6781	2.3	
0.75 wt.%	5	1.50	R1	1.6767	1.6741	2.6	4.1 ± 1.3 / 0.244 ± 0.078
			R2	1.6981	1.6931	5.0	
			R3	1.6931	1.6883	4.8	
0.75 wt.%	5	1.75	R1	1.6453	1.6340	11.3	10.9 ± 1.2 / 0.652 ± 0.056
			R2	1.6340	1.6244	9.6	
			R3	1.7343	1.7225	11.8	
0.75 wt.%	10	1.00	R1	1.4548	1.4496	5.2	5.2 ± 1.0 / 0.315 ± 0.065
			R2	1.7912	1.7869	4.3	
			R3	1.7869	1.7807	6.2	
0.75 wt.%	10	1.50	R1	1.6780	1.6725	5.5	5.4 ± 1.2 / 0.321 ± 0.070
			R2	1.7032	1.6966	6.6	
			R3	1.6966	1.6924	4.2	
0.75 wt.%	10	1.75	R1	1.6464	1.6413	5.1	6.5 ± 1.2 / 0.386 ± 0.069
			R2	1.6984	1.6909	7.5	
			R3	1.6909	1.6840	6.9	
1.00 wt.%	5	1.00	R1	1.4518	1.4488	3.0	3.7 ± 0.8 / 0.234 ± 0.034
			R2	1.6539	1.6494	4.5	
			R3	1.6494	1.6457	3.7	
1.00 wt.%	5	1.50	R1	1.4894	1.4855	3.9	4.2 ± 0.9 / 0.258 ± 0.053
			R2	1.6781	1.6729	5.2	
			R3	1.6729	1.6695	3.4	
1.00 wt.%	5	1.75	R1	1.4461	1.4411	5.0	5.2 ± 0.9 / 0.337 ± 0.053
			R2	1.6142	1.6080	6.2	
			R3	1.6080	1.6035	4.5	
1.00 wt.%	10	1.00	R1	1.4568	1.4506	6.2	8.2 ± 1.8 / 0.506 ± 0.077
			R2	1.6910	1.6812	9.8	
			R3	1.6812	1.6726	8.6	
1.00 wt.%	10	1.50	R1	1.4895	1.4815	8.0	8.3 ± 1.3 / 0.516 ± 0.076
			R2	1.6782	1.6685	9.7	
			R3	1.6685	1.6613	7.2	
1.00 wt.%	10	1.75	R1	1.4441	1.4302	13.9	12.6 ± 1.4 / 0.797 ± 0.150
			R2	1.6723	1.6596	12.7	

Formulation	Load (N)	Speed (m/s)	Rep.	m_i (g)	m_f (g)	Δm (mg)	Mean ± SD: Δm (mg) / mass loss (%)
			R3	1.6596	1.6485	11.1	
